# Filament Depolymerization Can Explain Chromosome Pulling during Bacterial Mitosis

**DOI:** 10.1371/journal.pcbi.1002145

**Published:** 2011-09-22

**Authors:** Edward J. Banigan, Michael A. Gelbart, Zemer Gitai, Ned S. Wingreen, Andrea J. Liu

**Affiliations:** 1 Department of Physics and Astronomy, University of Pennsylvania, Philadelphia, Pennsylvania, United States of America; 2 Graduate Program in Biophysics, Harvard University, Boston, Massachusetts, United States of America; 3 Department of Physics, Princeton University, Princeton, New Jersey, United States of America; 4 Department of Molecular Biology, Princeton University, Princeton, New Jersey, United States of America; UNC Charlotte, United States of America

## Abstract

Chromosome segregation is fundamental to all cells, but the force-generating mechanisms underlying chromosome translocation in bacteria remain mysterious. *Caulobacter crescentus* utilizes a depolymerization-driven process in which a ParA protein structure elongates from the new cell pole, binds to a ParB-decorated chromosome, and then retracts via disassembly, pulling the chromosome across the cell. This poses the question of how a depolymerizing structure can robustly pull the chromosome that disassembles it. We perform Brownian dynamics simulations with a simple, physically consistent model of the ParABS system. The simulations suggest that the mechanism of translocation is “self-diffusiophoretic”: by disassembling ParA, ParB generates a ParA concentration gradient so that the ParA concentration is higher in front of the chromosome than behind it. Since the chromosome is attracted to ParA via ParB, it moves up the ParA gradient and across the cell. We find that translocation is most robust when ParB binds side-on to ParA filaments. In this case, robust translocation occurs over a wide parameter range and is controlled by a single dimensionless quantity: the product of the rate of ParA disassembly and a characteristic relaxation time of the chromosome. This time scale measures the time it takes for the chromosome to recover its average shape after it is has been pulled. Our results suggest explanations for observed phenomena such as segregation failure, filament-length-dependent translocation velocity, and chromosomal compaction.

## Author Summary

Reliable chromosome segregation is crucial to all dividing cells. In some bacteria, segregation has been found to occur in a rather counterintuitive way: the chromosome attaches to a filament bundle and erodes it by causing depolymerization of the filaments. Moreover, unlike eukaryotic cells, bacteria do not use molecular motors and/or macromolecular tethers to position their chromosomes. This raises the general question of how depolymerizing filaments alone can continuously and robustly pull cargo as the filaments themselves are falling apart. In this work, we introduce the first quantitative physical model for depolymerization-driven translocation in a many-filament system. Our simulations of this model suggest a novel underlying mechanism for robust translocation, namely self-diffusiophoresis, motion of an object in a self-generated concentration gradient in a viscous environment. In this case, the cargo generates and sustains a concentration gradient of filaments by inducing them to depolymerize. We demonstrate that our model agrees well with existing experimental observations such as segregation failure, filament-length-dependent translocation velocity, and chromosomal compaction. In addition, we make several predictions–including predictions for the specific modes by which the chromosome binds to the filament structure and triggers its disassembly–that can be tested experimentally.

## Introduction

Several processes involved in DNA partitioning rely on depolymerization of filaments for translocation. In eukaryotes, depolymerizing microtubules [1] position chromosomes before cell division via macromolecular couplers and/or molecular motors bound to the microtubules [2], [3]. In prokaryotes, however, no such coupler or motor has been identified. Instead, proteins bound to the chromosome or plasmid bind directly to filaments and trigger their depolymerization [4], [5]. This poses the question of whether in the absence of a coupler, DNA can be pulled in a robust fashion, without becoming detached from the filaments as they disassemble.

Type I low-copy-number-plasmids [6], [7], chromosome I of *Vibrio cholerae* [8], and the chromosome of *Caulobacter crescentus* [9]–[12] all share a common segregation mechanism that relies on pulling mediated by filament depolymerization. This conserved system relies on three central components: the ATPase ParA, the DNA-binding protein ParB, and a centromere-like DNA locus. ParA is a deviant Walker-type ATPase that upon binding ATP forms dimers that can polymerize and associate with DNA [10], [13]. ParB interacts with ParA directly and stimulates ATP hydrolysis, causing ParA to dissociate into free monomers [13]. The spatial and temporal organization of ParA and the ParB-binding *parS* chromosomal locus can lead to robust chromosome segregation *in vivo*. For example, in *C. crescentus*, the chromosomal origin (*ori*) is initially localized at a single cell pole (the “stalked” pole) [14], and must translocate to the opposite “swarmer” pole before cell division. In predivisional cells, approximately one thousand ParB are bound via *parS* near the origin of the chromosome (*ori*) [9], [15]. There appear to be several distinct stages of ParB-*parS*-*ori* complex translocation [11]; our focus is on the final, most rapid stage in which the complex binds to filaments of ParA and translates from partway across the cell to the swarmer pole at a velocity of 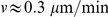 [9], [11], [16], [17]. As the ParA bundle depolymerizes, presumably due to ParB-induced ATP hydrolysis or nucleotide exchange [7]–[11], [13], [15], [18], [19], the ParB-*parS*-*ori* complex remains localized near the edge of the ParA structure [8], [10]–[12].

For eukaryotic chromosome segregation driven by depolymerization of microtubules [2], [3], models generally assume the existence of a “coupler” that attaches the chromosome to the depolymerizing microtubules. This coupler moves along the microtubule ahead of the depolymerizing end, either because it slides along it diffusively [20]–[24], because it is pushed by conformational changes near the tip of the microtubule [23]–[26], or because it has a complex internal structure that causes it to process [3], [27]. Of the existing models of bacterial chromosome segregation [28]–[33], only a few address the question of how depolymerizing proteins can cause translocation. Typically, these models attempt to explain ParAB partitioning systems with reaction-diffusion models or general thermodynamic arguments, but do not address the conditions required for robust translocation [31], [32].

**Figure 1:**
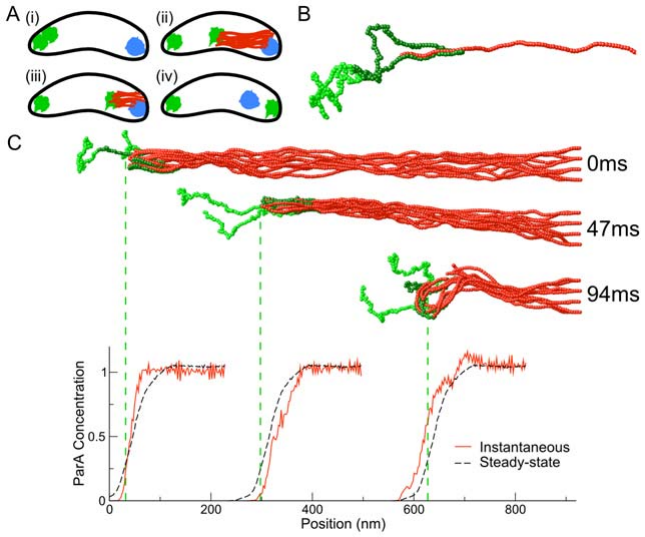
Schematic model for chromosome segregation and simulation snapshots. (A) Model of chromosome segregation in *Caulobacter crescentus*. (*i*) Initially, the two copies of the origin of replication (*ori* - green) and the terminus (*ter* - blue) of the chromosome are localized at the stalked and swarmer poles, respectively. (*ii*) The two origins separate and a structure of ParA protein (red) emanating from the swarmer pole comes into contact with the medial origin; ParB, polymerized on the chromosome near the origin, binds to ParA. (*iii*) ParB and the origin localize with the end of the ParA and move across the cell as ParA depolymerizes. (*iv*) The origin localizes near the swarmer pole; the terminus moves towards mid-cell. (B) Snapshot of ParB-ParA binding in simulation. The central strip of the ParB polymer (dark green) binds side-on to ParA filaments, whereas the peripheral segments of the ParB polymer (light green) cannot bind to ParA. (C) Snapshots of the full simulation and corresponding ParA filament concentration profiles (red). The dashed green lines indicate the center of mass of the ParB polymer. ParB binds to ParA and disassembles the ParA bundle (for clarity, depolymerized ParA monomers are not displayed). This interaction creates a steady-state ParA filament concentration gradient (black), which moves with and transports the ParB across the cell, providing a mechanism for chromosome segregation.

Here we ask whether depolymerization of ParA by ParB without a coupler is sufficient to explain the observed translocation in prokaryotic DNA partitioning. We performed Brownian dynamics simulations that explicitly incorporate the biochemistry of the primary constituents of the ParABS segregation system. In our simulations, a polymer representing the ParB-*parS*-*ori* complex (henceforth referred to as the “ParB polymer”), binds to a filamentous ParA bundle and initiates disassembly of ParA. We find that the ParB polymer can indeed exhibit robust, depolymerization-driven translocation via a novel mechanism (Fig. 1), provided certain conditions are met.

## Results

### Simulating ParB translocation

To understand the mechanism by which ParA translocates ParB, we performed Brownian dynamics simulations of a ParB polymer interacting with an anchored ParA filament bundle (Fig. 1c). The ParB polymer, shown in Figs. 1b–c, corresponds to the ParB-*parS*-*ori* complex. It is represented by a semi-flexible chain of monomeric subunits, typically of length 100 subunits. The center section (dark green in Fig. 1b), typically of length 50 subunits, represents the part of the chromosome that binds to ParA via ParB, while the two peripheral segments (light green in Fig. 1b only) cannot bind to ParA.

During robust translocation, the ParB polymer remains localized near the tip of the ParA bundle and moves across the cell (see snapshots in Fig. 1c and Video S1). By inducing disassembly, ParB creates a concentration gradient of ParA filaments that remains fixed with respect to the center of mass of the ParB polymer. Thus, the ParA concentration profile translocates with the ParB, and exhibits only small, short-lived fluctuations around a well-defined steady-state mean (Fig. 1c).

### Translocation is most robust when ParB binds side-on to ParA

Since the precise nature of the ParB–ParA interaction is unknown, we used our simulations to identify the modes of binding and disassembly that provide robust translocation. In our model (see Methods), ParB binds to ParA subunits in the filament bundle (Fig. 1c). The ParB polymer hydrolyzes ParA subunits that it binds to; once a subunit at the tip of a ParA filament is hydrolyzed, it can depolymerize from the filament. Monomers rapidly diffuse away once they have depolymerized. Some interaction/disassembly mechanisms or parameter ranges lead to robust translocation of the ParB polymer, while others lead to failure by rapid detachment:

#### Tip-only binding

**Figure 2:**
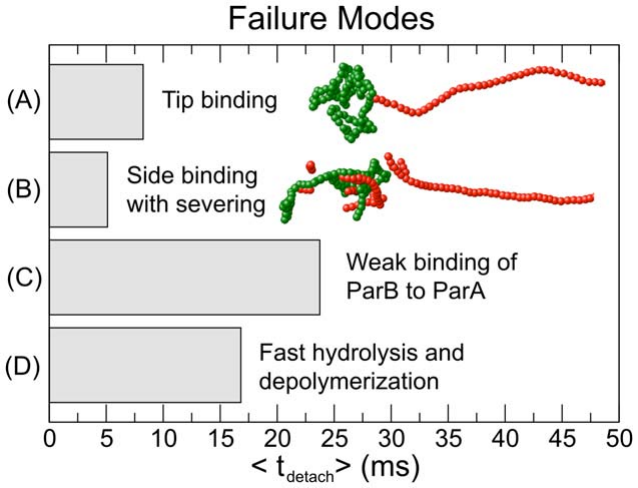
Mean time, 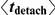, to first detachment of ParB polymer from ParA for various failure modes. In a standard simulation, ParB binds ParA filaments side-on, and hydrolyzes individual ParA subunits. Hydrolyzed ParA disassembles from the tip of each filament in the bundle. In a typical simulation it takes about 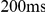 for the ParB polymer to translocate across a distance of 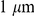. However, ParB completely detaches in a short time if (A) the ParB polymer binds to only the tips of the ParA filaments or (B) if ParA filaments disassemble via mid-filament severing. In addition, the ParB polymer detaches if (C) ParB binds too weakly to ParA or (D) ParA disassembles too quickly. Measurements for (A) and (B) are taken from simulations with side-binding with severing or tip binding, respectively, with standard parameters. Measurements for (C) and (D) are taken from simulations with the slowest disassembly rate or highest binding energy, respectively, for which the mean time to first detachment is shorter than the time required for the ParB polymer to translocate across the cell.

In this model, ParB binds only to the tips of ParA filaments (Fig. 2a). Since the number of ParA filament tips is limited, the ParB polymer is held only weakly to the ParA bundle, and small fluctuations can cause it to detach (Fig. 2a). In principle, this failure mode could be suppressed by increasing the number of ParA filaments within the bundle, but translocation is intrinsically fragile for this model.

#### Side-binding with filament severing

As an alternative, we allow ParB to bind to the sides of the ParA filaments (Fig. 1b). In this model, ParA filaments can disassemble by severing in addition to disassembling from the filament tips (Fig. 2b). Severing may occur at the location of any ParA subunit that has been hydrolyzed by ParB. Typically, we find that the ParB polymer binds to multiple severed ParA segments, preventing ParB from binding to the remaining filaments in the ParA bundle (Video S2). As a result, the ParB polymer rapidly detaches from the anchored bundle. ParA severing therefore does not lead to reliable ParB translocation.

#### Side-binding with tip-only disassembly

In this model, ParB binds side-on to ParA filaments (Figs. 1b–c) and ParA disassembles only at the filament tips. In this case, the ParB polymer translocates across the cell without detaching from the ParA bundle for a wide range of parameters. However, under certain extreme conditions, translocation fails:

#### Weak binding

If the ParB–ParA binding energy, 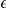, is too small, ParB quickly detaches from ParA due to thermal noise and the force from the rest of the ParB polymer (Fig. 2c).

#### Fast hydrolysis and depolymerization

Rapid detachment occurs if the ParA hydrolysis rate, 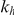, and ParA depolymerization rate, 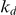, are both too large (Fig. 2d).

Our major result is that translocation is most robust in the side-binding model with disassembly only from the tips of ParA filaments. The rest of our simulations use this robust mode of disassembly and translocation, and henceforth, we refer to side-binding with tip-only disassembly as our standard model.

### The rate of disassembly controls the ParB translocation velocity

To understand how ParA translocates ParB, we identified variables controlling the translocation velocity, 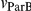. In all cases, we find that 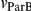 is given by the mean rate, 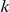, of disassembly of a ParA filament, so that 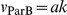, where 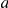 is the length of a ParA subunit. In order for a subunit to disassemble from the tip of a ParA filament, the subunit must bind to ParB, its ATP must hydrolyze, and the subunit must fall off. 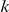 therefore depends on the distance, 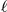, that the ParB polymer typically penetrates into the ParA bundle and causes ParA-ATP hydrolysis, the rate, 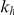, of ParA-ATP hydrolysis, and the rate, 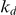, at which a ParA subunit depolymerizes once hydrolyzed.

In turn, the penetration length, 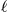, depends on the shape of the ParB polymer. In our simulations, the freely diffusing ParB polymer adopts an isotropic, globular equilibrium shape. The maximum value, 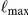, of the penetration length, 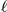, is achieved if the ParB polymer is able to maintain this equilibrium shape as it is pulled by ParA. If the disassembly rate, 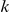, is too high, the ParB polymer is pulled along so rapidly that it does not have time to relax to its equilibrium shape. In this case, the ParA bundle pulls the leading region of the ParB polymer faster than the rear of the polymer can respond to the perturbation and the ParB polymer stretches out. Because the part of ParB polymer does not keep pace with the retraction of the depolymerizing ParA bundle, the ParB polymer does penetrate as deeply into the ParA bundle, so 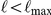.

**Figure 3:**
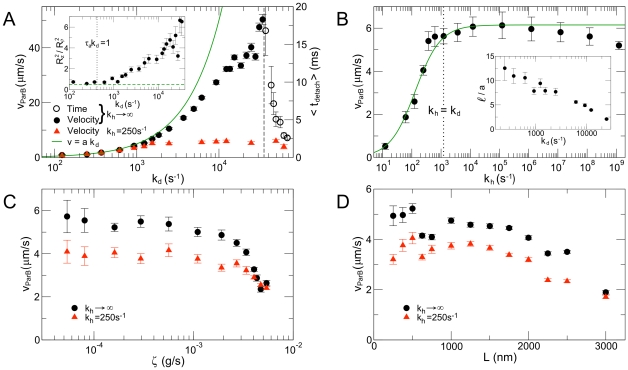
Dependence of translocation velocity on disassembly rate and relaxation time. (A) Translocation velocity, 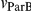 (solid symbols), increases with depolymerization rate, 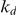. At low 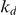, 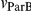 is linear in 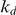, scaling as 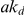 (green curve), where 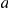 is the diameter of a ParA subunit. At large 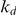, with an arbitrarily fast hydrolysis rate, 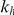, the ParB polymer detaches from the ParA bundle in an observably short time, 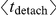 (open symbols). The dashed line separates the regime of translocation from the regime of detachment. For small 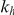 (red triangles), translocation velocity saturates at intermediate values of 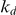 and 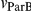. *Inset:* Ratio of the 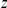-component of the radius of gyration of the ParB polymer squared to the 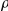-component squared (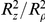). At large 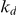, the polymer stretches along the axis of motility. The black dotted line marks the 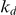 at which the depolymerization time, 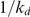, exceeds the effective relaxation time, 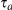 (Eq. 2), of the ParB polymer. The green dashed line indicates 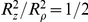, which is expected for an isotropic polymer coil. (B) 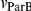 grows with hydrolysis rate for small 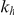 and saturates at 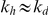 (indicated by dotted line). This behavior can be fit by 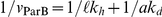 (green, see Eq. 3). *Inset:* Variation of the best-fit length scale, 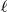, over ParA subunit diameter, 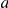, with 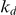. (C) 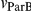 is insensitive to the total drag, 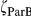, on the ParB polymer over several orders of magnitude for both fast 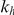 (black) and slow 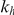 (red). For very large 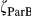, the ParB polymer translocates more slowly. (D) For a fixed quantity of ParB as one component of the polymer, longer polymers move more slowly than shorter polymers for both fast 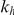 (black circles) and slow 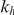 (red triangles). Unless noted to be varying, variables have the following values: 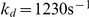, 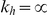 (black circles) or 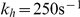 (red triangles), 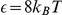, 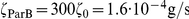, 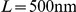, and there are 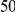 subunits that can bind to ParA in the ParB polymer. In (D), 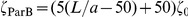.

We now estimate the time for the ParB polymer to relax to its equilibrium size. In our simulations, since ParB decorates the center section of the polymer and binds to ParA, the undecorated peripheral segments of the chain are the first ones to stretch out when the ParB polymer is pulled too rapidly (Video S3). The stretching of the peripheral segments is governed by the equation:
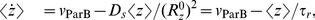
(1)where 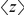 is the ensemble-averaged 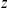-distance between the ends of a peripheral segment pulled by one end in the 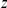-direction, 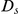 is the diffusion coefficient of the segment, 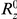 is the 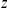-component of its equilibrium radius of gyration, and the relaxation time, 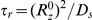, is the ratio of its internal drag, 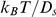, to the effective spring constant, 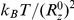 (see Text S1). Stretching is appreciable if 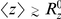, so for translocation in steady state (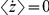), stretching becomes appreciable for 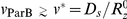, or, equivalently, 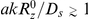 (inset to Fig. 3a). The shape of the pulled ParB polymer is therefore governed by the product 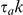, where we have defined
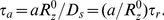
(2)


The penetration length, 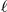, depends directly on the shape of the ParB polymer. For large 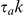 the ParB polymer is pulled rapidly and 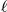 is small. This is because the ParB polymer is pulled away from the ParA bundle, leading to less overlap of the volume of the ParB polymer with the volume of the ParA bundle. As a result, there is less binding between individual ParB subunits with ParA subunits. As 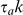 decreases, 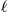 increases and saturates at 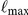 for 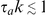 (inset to Fig. 3b). In the latter regime, the disassembly rate is 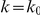, where
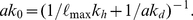
(3)Thus, the translocation velocity is controlled by the effective relaxation time, 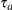, and the maximum disassembly rate 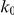.

### Three regimes of translocation velocity

We find that the translocation velocity, 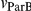, falls into three regimes, depending on 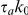:
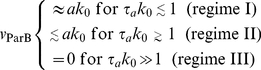
(4)For 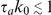 (regime I), the ParB polymer retains its equilibrium shape as it is pulled across the cell at the velocity 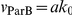. For 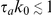 (regime II), the ParB polymer stretches as it is pulled and does not penetrate deeply into the ParA bundle. Since fewer ParA subunits bind to ParB, fewer are hydrolyzed and 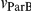 drops below 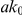. For 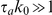 (regime III), the ParB polymer is so elongated that ParB binds to very few ParA subunits and the ParB polymer quickly detaches from the ParA bundle, leading to 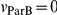.

This physical picture explains the results shown in Fig. 3, where we vary both the disassembly rate, 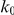 (Figs. 3a–b) and the effective relaxation time, 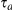 (Figs. 3c–d). Specifically, Fig. 3a shows how 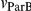 depends on the depolymerization rate, 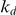. For the black circles in Fig. 3a, the hydrolysis rate, 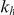, is effectively infinite so that 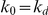 (Eq. 3). In this case, for sufficiently small 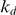, the system is in regime I and 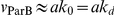. As 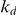 increases, 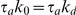 also increases; as a result, the ParB polymer stretches (inset to Fig. 3a) and the system crosses into regime II, where 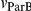 drops below 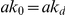. At very large 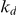, the system reaches regime III, and 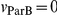.

In contrast, if 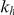 is small (red triangles in Fig. 3a), then 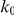 cannot exceed 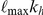 as 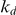 increases (Eq. 3). Therefore, for small 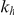, the ParB polymer remains in regime I, 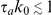, for all 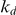, so that 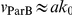 and translocation is robust for any 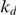. Thus, by decreasing the overall rate of disassembly by lowering 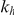, the system can achieve robust translocation, albeit at a cost to velocity.

Fig. 3b shows how 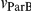 varies as 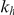 increases. In this case, 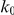 saturates to 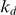 at large 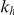 (Eq. 3). Since 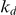 is chosen to be small, we find 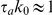 over the entire range of 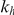, meaning the system is in regime I and 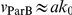.

The different velocity regimes can also be explored by varying 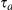 instead of 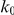. Fig. 3c shows that 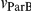 is insensitive to the total drag, 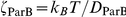, on the polymer when 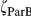 and thus 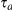 are small. In this case, 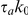 is small, and the system is in regime I. As 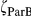 increases, 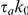 increases, causing 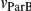 to drop below 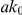 as the system crosses into regime II.

Fig. 3d shows the effect of the total contour length, 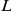, of the ParB polymer. For small 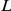, 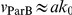 is constant since the system is in regime I. As 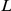 increases, 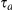 increases, a9nd when 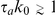, 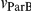 crosses into regime II and 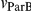 drops below 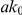.

### Dependence of the translocation velocity on binding energy, binding sites, applied load, and other physical variables

**Figure 4:**
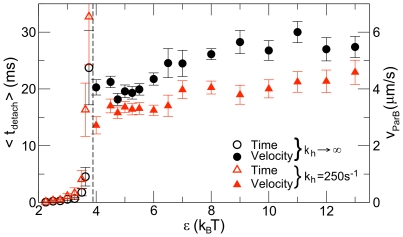
Dependence of translocation velocity on ParB–ParA binding energy. ParB detaches in an observably short time, 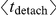, when the binding energy, 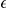, is too small (open symbols). When 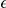 is large enough, 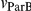 (solid symbols) is non-zero, and is insensitive to 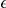 over the observed range for both fast (black) and slow (red) 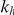. The dashed line separates regimes of detachment and translocation.

Fig. 4 shows that 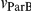 has a threshold dependence on the ParB–ParA binding energy, 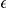. As shown in Figs. 2c, 4, ParB rapidly detaches from the ParA bundle if 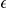 is too small. However, as long as 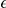 is sufficiently large, the ParB polymer remains attached to the bundle throughout the simulation and translocates with a velocity that is insensitive to 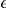 and is set by 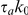 (Eq. 4). We observe similar behavior as the number of binding sites on the ParB polymer is varied. If there are too few binding sites, the ParB polymer quickly detaches from ParA. Above a threshold value, however, 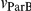 does not sensitively depend on the length of the binding strip (Fig. S1). The translocation velocity is also insensitive to the filament density within the ParA bundle, the arrangement of filaments in the bundle, and stiffness of the ParB polymer (Figs. S2, S3, S4). Finally, we have also verified that our main results hold when the form of the ParB–ParA binding potential is altered to allow binding by multiple points on ParB and/or ParA subunits.

### Detachment force for the ParB polymer

We next investigate the extent to which motility is robust to an external force on the ParB polymer that opposes translocation. The external force, 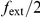, opposes translocation by pulling on each end of the ParB polymer. In our simulations, we find that 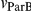 is unperturbed for 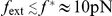 (Fig. S5). For 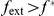, however, the ParB polymer rapidly detaches from the ParA bundle and translocation stalls.

In order to understand this behavior, we analytically estimate the “detachment force,” 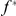, required to pull the ParB polymer off of the ParA bundle in a time, 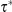, that is approximately equal to the time required for the ParB polymer to translocate across the cell (see Text S1 for details).

In our simulations, we model the ParB-*parS*-*ori* complex as a polymer chain comprised of 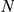 monomeric subunits. Each subunit in the central strip of the ParB polymer binds with a binding energy, 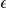, to a subunit in the ParA bundle. Thus, the total strength of the attraction between the ParB polymer and the ParA bundle is approximately proportional to 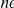, where 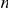 is the number of ParB subunits actually bound to ParA. Since ParB subunits lie in approximately a Gaussian distribution about the center of mass of the ParB polymer [34], 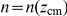, depends on the location, 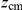, of the center of mass of the ParB polymer.

Now consider the effect of a force 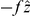 on the ParB polymer that opposes translocation in the 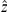 direction. At the simplest level, based on the above analysis, the ParB polymer may be replaced by a point particle at the center of mass of the ParB polymer, 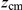, in an effective potential given by
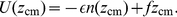
(5)The first term is due to ParB binding to ParA and the second term is the work done by the external pulling force, 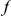. As 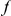 increases, the minimum of 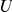 shifts to lower values of 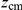 and the number of bound ParB sites decreases, eventually leading to unbinding of the ParB polymer from the ParA bundle.

The mean time for the particle to escape from the potential well (to detach from the ParA bundle) is well approximated by the Kramers escape time, 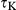 for this potential [35], [36]:
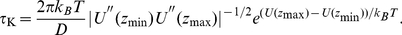
(6)Given these expressions, we calculate the detachment force 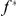 to be the force 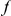 for which the escape time, 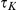, is equal to 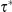, the time required for the ParB polymer to translocate across the cell.

In simulations with our standard model, the central binding strip has 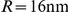 and 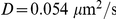. There are 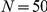 ParB subunits that bind to ParA with energy, 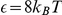, so the maximum total binding energy is 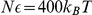. The ParB polymer translocates at 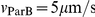, so that the time to translocate 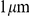 is 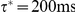. With these parameters, we estimate that the detachment force is 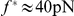. An estimate for the detachment force under more realistic conditions (*in vivo*) is given in the Discussion section.

This order of magnitude estimate agrees with our simulations at high depolymerization rates, 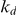 (Fig. 3a), large drag coefficients, 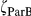 (Fig. 3c), and large external pulling forces, 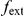 (Fig. S5). In the first case, the mean time to first detachment is shorter than the translocation time for 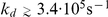; this suggests that the force, 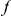, required for rapid detachment is 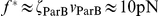. Similarly, we find that the ParB polymer fails to translocate for 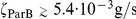, giving a detachment force of 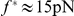. In addition, we have conducted simulations in which we apply an external force, 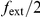, to each of the ends of the polymer. For these simulations, we find robust translocation up to a detachment force of 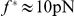.

### The ParB polymer translocates even when the ParA bundle is not anchored

So far, we have assumed that the ParA bundle is anchored to the pole. Recent reports suggest that in *C. crescentus*, ParA is localized to the swarmer pole by TipN [10], [12], but it is unclear if TipN actually anchors ParA. We therefore examined whether ParB translocation could occur if the ParA bundle is localized but not anchored.

**Figure 5:**
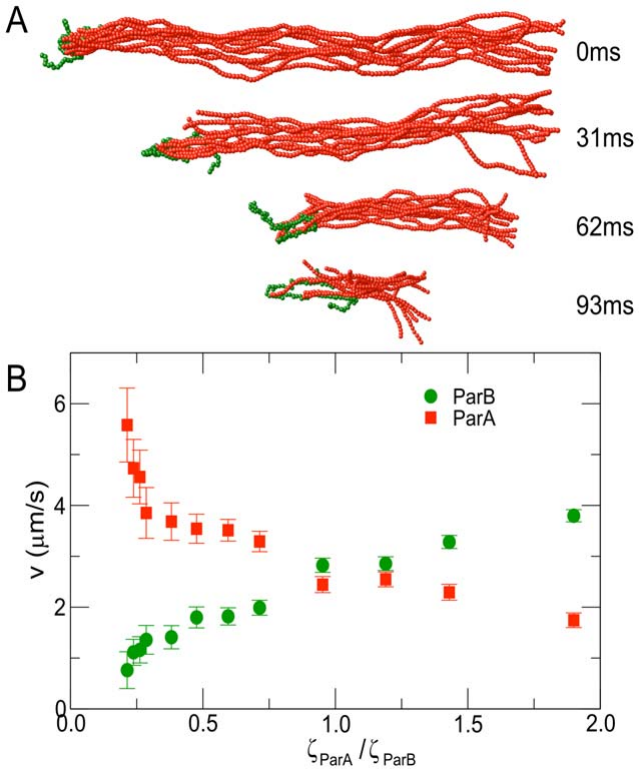
The ParB polymer translocates even when the ParA bundle is unanchored. (A) Snapshots of a simulation in which the ParA bundle is not anchored at its right end (swarmer pole). The ParA bundle (red) is pulled towards mid-cell as the ParB (green) moves towards the swarmer pole. (B) Dependence of speeds of ParA (red) and ParB (green) on the ratio of drags, 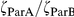. In these simulations, 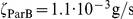 and 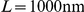.

Fig. 5 shows that the ParB polymer translocates even when the ParA bundle is unanchored. We understand this through Newton's third law, which dictates that the force, 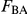, that pulls ParB to ParA is equal in magnitude but opposite in direction to the force on ParA. Thus ParB is pulled towards the swarmer pole while ParA is simultaneously pulled away from it:
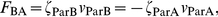
(7)

where 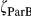 and 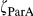 are the drag coefficients of the ParB polymer and ParA filament bundle, respectively.

In the case of a long, unanchored ParA bundle, 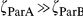 and the ParB polymer translocates across the cell while the ParA bundle remains relatively stationary (Fig. 5b). However, if the ParA bundle is sufficiently small (*e.g.*, when the ParB has nearly reached the swarmer pole), 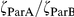 is small, so the large ParB polymer remains relatively stationary while pulling the smaller, disassembling ParA bundle towards mid-cell (Fig. 5b).

## Discussion

Based on recent experimental observations [7]–[12], [15], we have tested several simulation models (Fig. 2) and discovered a robust mechanism for chromosome segregation in *C. crescentus* via the ParABS system.

### Self-diffusiophoresis can explain ParA pulling

Our simulations point to a specific physical mechanism underlying translocation in the ParABS system. We find that disassembly of ParA generates a steady-state ParA filament concentration gradient that remains fixed in the center-of-mass frame of the translocating ParB polymer (Fig. 1c). In other words, disassembly of ParA allows the ParA filament concentration gradient to translocate with the particle across the cell so that at all times the ParB polymer is moving up the concentration gradient of ParA to satisfy its attraction to ParA. Our simulations do not include fluid flow, but it is known that external concentration gradients can also drive motion of a particle in a fluid environment; the latter phenomenon is known as “diffusiophoresis.” If the particle (in this case, the ParB-*parS*-*ori* complex) is attracted to the solute (the ParA filament bundle), it will translocate up the concentration gradient towards high solute concentrations [37]. In “self-diffusiophoresis,” the particle itself (the ParB-*parS*-*ori* complex) generates and sustains the solute concentration gradient [38], [39] via disassembly of ParA. We emphasize that ParB-induced depolymerization (particle-induced destruction of solute) is central to this process. Without depolymerization, the ParA bundle would remain intact and the concentration of ParA filaments would not change with time. As a result, the ParA concentration profile would not be able to move with the particle and translocation would not occur.

This intrinsically many-body mechanism is distinct from biased diffusion. In contrast to biased diffusion mechanisms which apply to a coupler that attaches a load to a single filament or fiber [20]–[22], [26], self-diffusiophoretic translocation can occur even if the ParB polymer does not diffuse, as long as the ParB-ParA interaction range is finite. In self-diffusiophoresis, “diffusio” refers not to diffusion of a coupler, but to the key role of the solute gradient, just as the prefix in “electrophoresis” refers to an electric potential gradient [37]. The self-diffusiophoretic mechanism also differs from ones involving motion of a coupler [3], [20]–[27]; in our case, the load is not attached to a coupler that cannot detach from the depolymerizing filaments. Instead, the load is attached directly to the depolymerizing filaments via many non-permanent bonds.

It has been suggested that polymerization-driven motility, as in the case of F-actin in the lamellipodium of eukaryotic cells, also constitutes an example of self-diffusiophoretic motility [40], [41]. In that case, the object to be moved (*e.g.*, the cell membrane) is repelled by the structure (the branched actin network) that it builds in order to move. In depolymerization-driven translocation, on the other hand, the object to be moved (the ParB-DNA complex) is attracted to the structure (ParA) that it destroys in order to move.

The self-diffusiophoretic mechanism suggests modes of failure for translocation. For example, overexpression of ParA leads to segregation defects, and it has been suggested that these defects arise due to the increase in the quantity of delocalized ParA [12], [15]. This effect may be analogous to what we observe in our simulations with severing (Video S2), where instead of binding to the ParA bundle, ParB can bind to severed ParA filaments. This disrupts the steady-state generation of a translating ParA concentration gradient so that it does not support steady-state ParB polymer translocation. Similarly, when ParA is overexpressed, extra ParA monomers or protofilaments may diminish or erase the ParA concentration gradient created by depolymerization. Alternatively, the extra ParA could saturate ParB, preventing translation of the ParA gradient.

### Translocation is most robust for side-binding of ParB to ParA with disassembly only from the tip

We observe robust translocation over a wide range of physical parameters only if ParB binds to the sides of ParA filaments, triggering disassembly only from the tips of filaments (Fig. 1b–c). If ParB binds only to the tips of filaments, translocation is far less robust for two reasons. First, there are many fewer ParA subunits to which ParB can bind so the overall attraction between ParB and ParA is weaker. Second, the ParB polymer is localized near the tip of the bundle, at the very edge of the concentration gradient of ParA that drives translocation. In contrast, in the side-binding model, the ParB polymer penetrates further into the bundle so that it is localized near the steepest, central section of the concentration gradient (Fig. S6). Thus, in the tip-binding-only model, the ParB polymer is much more likely to detach from the ParA bundle due to thermal noise (Fig. 2a). This failure mode can only be averted by greatly increasing the binding energy or the number of filaments, and thus tips, in the ParA bundle.

We also find that ParA disassembly via severing does not provide robust translocation (Fig. 2b) because severed protofilaments can bind to ParB, reducing the attraction between the ParB polymer and the main ParA bundle, leading to detachment.

We therefore predict that ParB binds to the sides of ParA filaments and ParA filaments disassemble primarily from the tip. This prediction can be tested with *in vitro* experiments.

### Comparisons with experiments on Par-mediated chromosome pulling

Our model is sufficiently versatile to account for a range of experimental observations. For example, by varying the initial density and cross-linking of the ParA filament bundle in our simulations, we find cases in which some ParA filaments remain partially assembled even though the ParB polymer has translocated across the cell (Fig. S7). This is in agreement with the observations of Ptacin *et al.* [10], who found that in some cases, a fiber of ParA extended across the predivisional cell after *ori* had translocated.

We find that the robustness of translocation is primarily controlled by the quantity 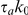, the product of an effective relaxation time (Eq. 2) and the maximum rate of disassembly of ParA (Eq. 3). The underlying details of the ParB polymer are only important insofar as they affect quantitative results such as the precise value of the relaxation time; they do not affect the qualitative physical principles described above.

Specifically, if 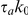 is too high, the ParB polymer stretches out and can detach from the ParA bundle. This finding suggests a possible role for chromosome organizing factors such as the SMC protein [14], [42]. In order to translocate reliably and efficiently, the chromosome of four million base pairs [14], [16] must be organized such that it does not overload the pulling mechanism. We propose that one important physical function of chromosomal organization and condensation is to minimize the effective relaxation time, 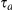, so that the chromosome can keep up with the retracting ParA bundle, to ensure robust translocation.

In addition, we find that the velocity is simply the product of the ParA subunit length and the maximum disassembly rate, 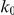, provided disassembly is slow enough to guarantee that 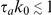 (Eq. 4). From the observed *ori* translocation velocity, 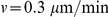 [9], [11], [16], [17], we estimate the *in vivo* ParA disassembly rate to be 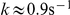, which is slower than the measured disassembly rate of dynamically unstable ParM filaments [43], but comparable to the disassembly rate of actin filaments [44].

The translocation velocity in our simulations is considerably higher, typically several 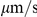, because we used high disassembly rates. Translocation is robust in our simulations at these high values of 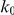 because the effective relaxation time, 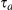, of our ParB polymer is fairly short. In the real system, where the effective relaxation time of the chromosome is likely to be considerably longer, it could be a biological necessity that both ParA disassembly and *ori* translocation proceed at slower than the simulated rates.

Likewise, in our simulations the ParB polymer detaches when it is pulled with a force 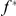 of order tens of pN, but this detachment force is likely to be much higher in the real system. The most important difference between our simulations and the actual bacterium lies in the number of ParB binding sites 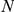. To estimate the detachment force, 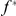, under realistic conditions, we first estimate 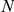, the maximum possible binding energy 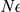, the extent of the chromosome 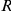, and the diffusion coefficient of the chromosome 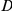. We first estimate 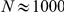 by assuming that ParB decorates the approximately 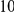 kilobase segment of the chromosome that was found to be the site of force exertion during translocation in [9]. For 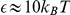 we therefore obtain a maximum binding energy of 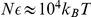. For ideal polymer chains [34], 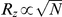 and 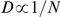. Thus, we estimate 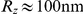 and 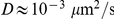. This crude estimate of 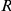 actually agrees well with experimental snapshots of *C. crescentus* during chromosome segregation [10], [11]. The estimate of 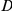 falls within the range 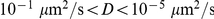, which is measured in *E. coli* for DNA segments of varying sizes [45], [46]. We note that 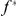 is insensitive to 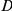, and varies by less than 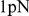 over that range.

According to experiments [9], [11], [16], [17], the ParB-*parS*-*ori* complex translocates across the cell in about 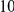 minutes. Using Eq. 6, we find that the detachment force is 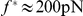. This value is of the same order of magnitude as the 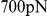 stall force for chromosome segregation along kinetochore fibers in eukaryotes [47], [48]. Thus, this estimate suggests that the mechanism we have proposed is both physically reasonable and biologically relevant.

### Implications for other phenomena

Insights from our results may extend to plasmid segregation by ParAB. In *Escherichia coli*, the ParA concentration profile is known to oscillate as plasmid pB171 is partitioned [6], [19], [49]. This dynamic behavior appears to be required for proper plasmid partitioning [7], [19]. We suggest that ParB creates a moving ParA filament concentration gradient that pulls the plasmid along as ParA disassembles.

In addition, our findings suggest an alternative explanation for observations that the distance that plasmid pB171 translocates in a given time interval increases approximately linearly with the initial ParA filament length [7]. Ringgaard *et al.* [7] suggest that this effect arises from a ParA filament-length-dependent plasmid detachment rate. However, we have shown that the relative velocities of the ParB polymer and the ParA bundle depend on the ratio of the viscous drags on ParA and ParB, 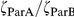 (Fig. 5). Thus, the observed dependence of plasmid translocation distances and velocities on ParA filament length may simply be a result of Newton's third law, due to the variation of 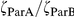 with ParA filament length.

Our simulations with unanchored ParA filaments suggest a new possibility for the mechanism of terminus segregation in *C. crescentus*. As translocation begins, the ParA filaments are long, so 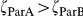 and the ParB polymer is pulled rapidly towards the swarmer pole. However, as the ParB polymer nears the swarmer pole the ParA filaments are much shorter and 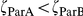 may be satisfied, so that the ParA bundle is pulled toward mid-cell. Experiments have indicated that ParA binds non-specifically to DNA [7], [10], [18]. Thus, we propose that DNA near the terminus is non-specifically bound to ParA and translocates away from the swarmer pole as ParA filaments are pulled toward mid-cell by the ParB-*parS*-*ori* complex. In contrast to previously suggested passive mechanisms [16], [30], [33], this is an active process, directly linked to *ori* translocation.

Our results provide a new paradigm for understanding depolymerization-driven translocation in prokaryotic DNA segregation systems. Since self-assembly and disassembly are ubiquitous in cellular systems, the creation of concentration gradients by these processes provides a general and robust mechanism for translocation.

## Methods

At the start of each simulation, ParA monomeric subunits form a cross-linked bundle of filaments. The ParB-decorated chromosome is represented by a semi-flexible chain of monomeric subunits, typically of length 100 subunits, divided into three sections. The center section, typically of length 50 subunits, represents the part of the chromosome bound to ParB; these subunits can bind specifically to ParA subunits. The two end sections of the ParB polymer flanking the ParB section do not bind to ParA.

### Biochemistry

The process of ParA disassembly begins when a ParB subunit binds to a ParA-ATP subunit. If the interaction energy, 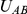, exceeds a certain threshold, 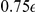, the ParA-ATP hydrolyzes at rate 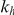. Once the ParA subunit hydrolyzes, it may detach from the ParA filament by depolymerization at rate 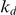 (after which it continues to interact with other subunits by the interaction 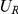). In our standard model, ParB binds to the sides of ParA filaments, and a hydrolyzed ParA subunit can only depolymerize if it is located at the tip of a ParA filament.

### Units

Simulation units are converted into physical units by taking the subunit length to be 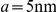. The typical subunit diffusion coefficient is taken to be 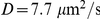, as measured in [50], and the diffusion coefficient for a particular subunit is 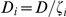 (typically 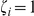 or 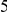, see below), giving a cell viscosity 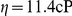 and a characteristic time scale 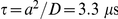. Typical runs are approximately 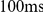 and simulation steps are 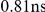.

### Interactions

Several interactions are included in the model; their specific forms are given below. All subunits are spheres with diameter 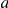 that repel each other if they overlap:
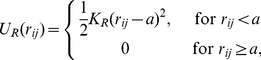
(8)where 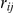 is the center-to-center distance between subunits 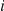 and 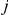 and 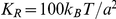. Within a ParA or ParB polymer chain, neighboring subunits are held together through an attractive harmonic potential:
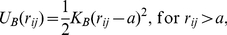
(9)with 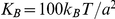. In order to hold the ParA bundle together, we typically take 40% of ParA subunits to be cross-linked to a subunit in a nearby filament through an attractive potential:
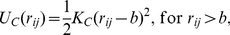
(10)where 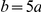 is the initial spacing of filaments in the ParA bundle and 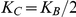. ParA filaments are stiffened by a bending potential [51]:
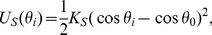
(11)where 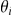 is the angle between the bond vector, 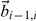, between ParA subunits 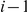 and 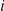, and the bond vector, 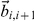, between subunits 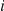 and 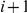. Thus, 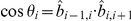, where 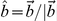. We take 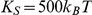 and 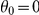. Similarly, the stiffness of the ParB polymer can be controlled by an interaction potential of form of Eq. 11 (however, in our standard model, 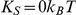 in the ParB polymer).

In addition, we introduce interactions so that binding between ParA and ParB occurs in specific spatial locations on the spheres representing the subunits. Each subunit 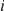 has a unit polarization vector, 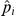, that determines the location of the binding site for the ParB–ParA interaction, and the following interaction potential aligns it to be at an angle 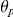 to the bond vectors 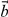 connecting adjacent subunits:
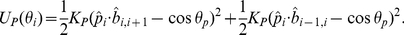
(12)We choose 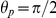 so that 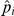 tends to be perpendicular to the bond vectors, and fix 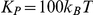 for ParA filaments and 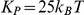 in the ParB polymer, which is relatively more flexible. Binding sites are arranged helically on the ParA filaments and the ParB polymer due to two additional interaction potentials. The first constrains polarization vectors on nearest-neighbor subunits on a given chain:
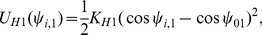
(13)where 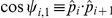 and 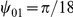 sets the pitch of the helix. Here, 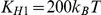 for ParA and 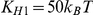 for ParB. The second potential has the same form,
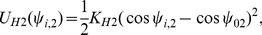
(14)but constrains polarization vectors on the next-nearest-neighbor subunits with 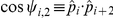 and 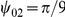. Here, 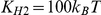 in ParA and 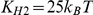 for ParB. Note that in addition to regulating the locations of the binding sites, Eqs. 13 and 14 implicitly regulate torsion within the ParB polymer.

Finally, ParB binds to ParA with a site-specific, short-ranged interaction potential:
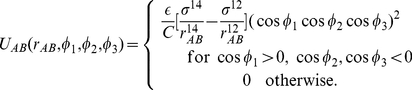
(15)where 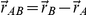 is the vector distance between the ParA and ParB subunits and 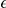 is the binding energy. In our standard model, 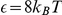. The normalization factor 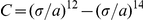 ensures that 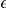 is the relevant energy scale for binding. The distance 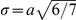 sets 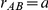 as the minimum of the binding potential. Binding site specificity is implemented through regulation of the angles between the polarization vectors on the ParA and ParB subunits as well as 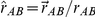. In Eq. 15, 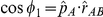, 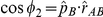, and 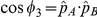. Binding is strongest when the two polarization vectors point towards each other and along 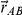.

We have also studied several variations of these models. For example, in a separate set of simulations, we set 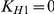 and 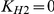 for both ParA and ParB, so that the binding sites were not arranged helically on the ParA filaments and ParB polymer. The orientation of the polarization vectors was set by 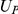, where 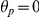 for tip binding and 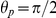 for side-on binding. We also studied cases in which monomeric ParB subunits did not possess specific orientations (polarization vectors). In these cases, ParA polarization vectors were set by 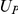, where 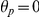 for both tip-binding and side-binding. Binding only weakly depended on the orientation of the ParA-ParB bond through a modified version of 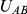, which we denote 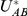 and 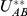 for tip-binding and side-binding, respectively. For tip-binding without ParB polarization vectors:
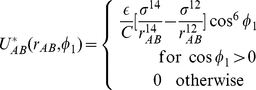
(16)For side-binding without ParB polarization vectors::
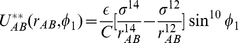
(17)where 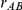, 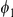, 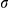, and 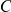 are as defined above.

### Equations of motion

All subunits in the system translate and rotate according to Brownian dynamics [52]. Thus, we solve a system of coupled Langevin equation where the velocity of each subunit is governed by the forces exerted by other subunits in the system as well as thermal forces, 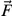 from the surrounding liquid medium:
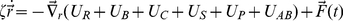
(18)

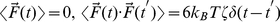
(19)and
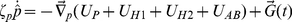
(20)

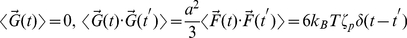
(21)The subunit friction constant is 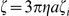, where 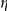 is the viscosity, and 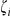 is a constant that determines the relative magnitude of the drag on subunit 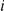. Typically, 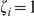 for ParA and normal ParB subunits, and 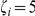 for ParB subunits that cannot bind to ParA. 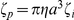 is the rotational friction coefficient.

## Supporting Information

Figure S1
**Behavior of the ParB polymer as a function of the length of the central ParB strip that binds to ParA.** If too few of the ParB can bind to ParA, the ParB polymer detaches in an observably finite average time, 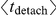 (open symbols). When the percentage of binding sites is above threshold, the translocation velocity, 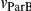, is non-zero. If there are enough binding sites to cause disassembly at all of the ParA filament tips simultaneously, 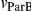 is insensitive to the number of ParB that can bind ParA. The dashed line separates the regimes of detachment and translocation.(PDF)

Figure S2
**Dependence of translocation velocity,** 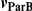 **, on the density of ParA filaments within the ParA bundle.** For ParA bundles of equal diameter, 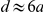, but different numbers of ParA filaments, the translocation velocities are approximately equal. Thus, 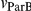 is insensitive to the density of filaments in the ParA bundle.(PDF)

Figure S3 **Snapshots of a simulation with a “ParA tube”.** The ParA filaments in the ParA bundle are arranged cylindrically. The snapshots are slightly rotated into the page and the thin black circle indicates the base of the cylinder. Translocation of the ParB polymer is insensitive to whether the ParA filaments are arranged as a tube or as a bundle. Depolymerized ParA monomers are not shown.
(PDF)

Figure S4
**Dependence of translocation velocity,** 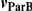 **, on the stiffness of the ParB polymer.** In our standard model, the ParB polymer is flexible, and the bending stiffness is 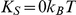. In order to simulate a stiff ParB polymer, we apply the bending potential in Eq. 11 to the ParB polymer. 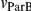 is insensitive to the bending stiffness over the observed range of 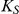.(PDF)

Figure S5
**Force-velocity relation for ParB polymer translocation in our simulations.** In these simulations, an external force, 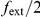, pulls on each of the two ends of the ParB polymer, thus opposing depolymerization-driven translocation. Translocation of the ParB polymer is unperturbed when subjected to external pulling forces up to 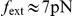.(PDF)

Figure S6
**Steady-state ParA concentration profiles for tip-binding-only and side-binding models.** Steady-state ParA concentration is plotted versus position relative to the center of mass of the ParB polymer, which is located at 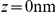 and indicated by the dotted green line. When ParB binds only to the tips of ParA filaments, the center of mass of the ParB polymer (dotted green line) localizes near the edge of the ParA filament concentration gradient (dashed black curve). This enables the ParB polymer to easily escape the ParA concentration gradient and detach from the ParA bundle due to thermal noise. However, when ParB can bind to the sides of ParA filaments, the ParB polymer penetrates further into the ParA bundle, and thus the center of mass (green) of the ParB polymer is localizes near the center of the ParA concentration gradient (dashed red curve). Thus, the ParB polymer is not susceptible to falling out of the ParA gradient and detaching from the ParA bundle due to thermal noise.(PDF)

Figure S7
**Snapshots of a simulation in which several ParA filaments remain after the ParB polymer has translocated.** If the initial spacing, 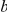, of the ParA filaments in the bundle is large, the ParB polymer may translocate by disassembling some, but not all, of the ParA filaments. In the snapshots shown, the initial ParA filament spacing is 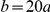, four times greater than the initial spacing, used in our standard simulations. This simulation demonstrates the versatility of our model by replicating one of the observations of Ptacin *et al.* (2010) [10]. This result can also be obtained with closely packed (*e.g.*, 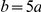) ParA filaments if the filament bundle contains a large number of filaments.(PDF)

Text S1
**Polymer relaxation time and estimated detachment force for the ParB polymer.** This text explains how to calculate the characteristic polymer relaxation time, 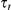, and the peripheral segment diffusion coefficient, 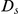. In addition, we provide details for the estimation of the detachment force, 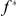.(PDF)

Video S1 **A movie of translocation of the ParB polymer in our standard simulation conditions.** The ParB polymer remains localized near the tip of the ParA bundle and translocates as the ParA bundle disassembles. Depolymerized ParA monomers are not shown.
(MOV)

Video S2 **A movie of a simulation run for the model in which ParB binds to the sides of ParA filaments and severs them.** The ParB polymer translocates briefly until severed ParA protofilaments bind to the ParB polymer and disrupt its binding to the main ParA filament bundle.
(MOV)

Video S3
**Stretching of the ParB polymer.** When the maximum ParA disassembly rate, 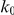, is sufficiently large, the ParB polymer does not have time to relax to its equilibrium shape as it is pulled, and therefore stretches out. The ParB polymer consists of three segments; the two peripheral segments (light green), which cannot bind to ParA, and the central segment (dark green), which can bind to ParA. Note that the peripheral segments of the ParB polymer stretch, while the central segment of the ParB polymer is initially bound to the ParA filament bundle. When the peripheral segments stretch too far, they start to stretch the central segment, thus decreasing the length, 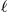, that the ParB polymer penetrates into the ParA bundle. This can lead to detachment for sufficiently high 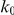. In this movie, 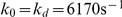, five times greater than the ParA disassembly rate, 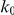, in our standard simulations (see Fig. 3 caption).(MOV)
