# Supplementary figures and images for: Filament Depolymerization Can Explain Chromosome Pulling during Bacterial Mitosis
Source: PLoS Comput Biol. 2011 Sep 22;7(9):e1002145. doi: 10.1371/journal.pcbi.1002145 (PMC3178632; doi:10.1371/journal.pcbi.1002145)

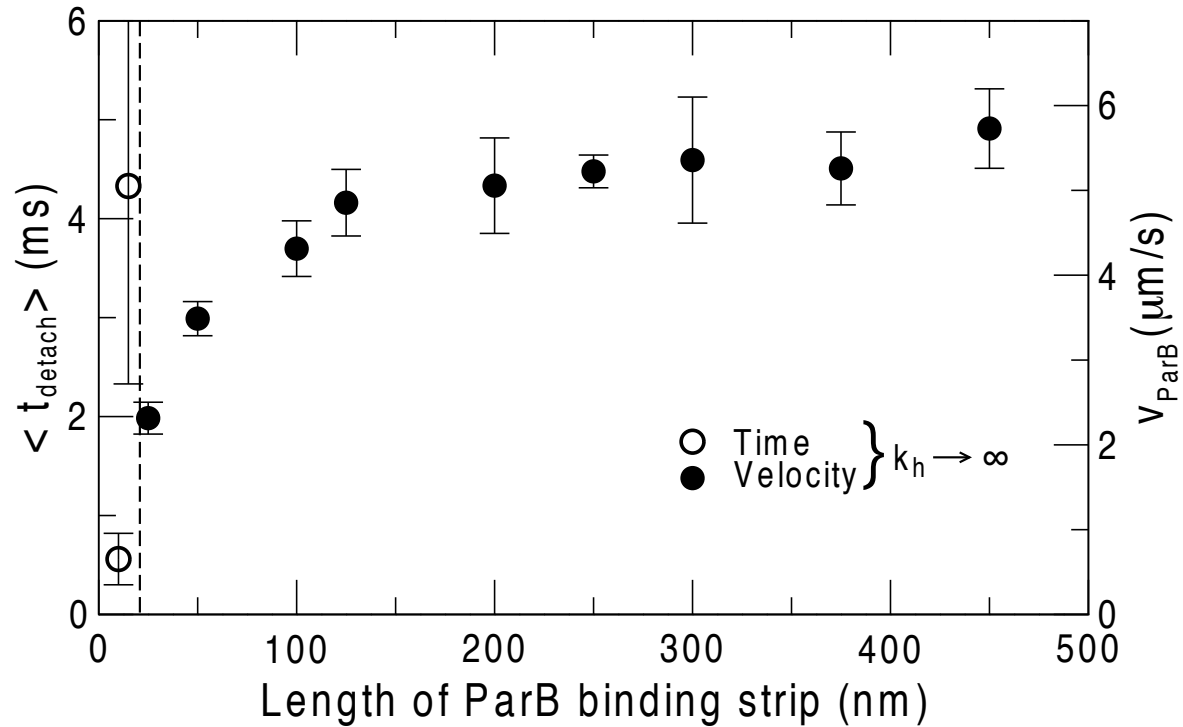

Supplement: Figure S1 — Behavior of the ParB polymer as a function of the length of the central ParB strip that binds to ParA. If too few of the ParB can bind to ParA, the ParB polymer detaches in an observably finite average time, (open symbols). When the percentage of binding sites is above threshold, the translocation velocity, , is non-zero. If there are enough binding sites to cause disassembly at all of the ParA filament tips simultaneously, is insensitive to the number of ParB that can bind ParA. The dashed line separates the regimes of detachment and translocation. (PDF) [file pcbi.1002145.s001.pdf]

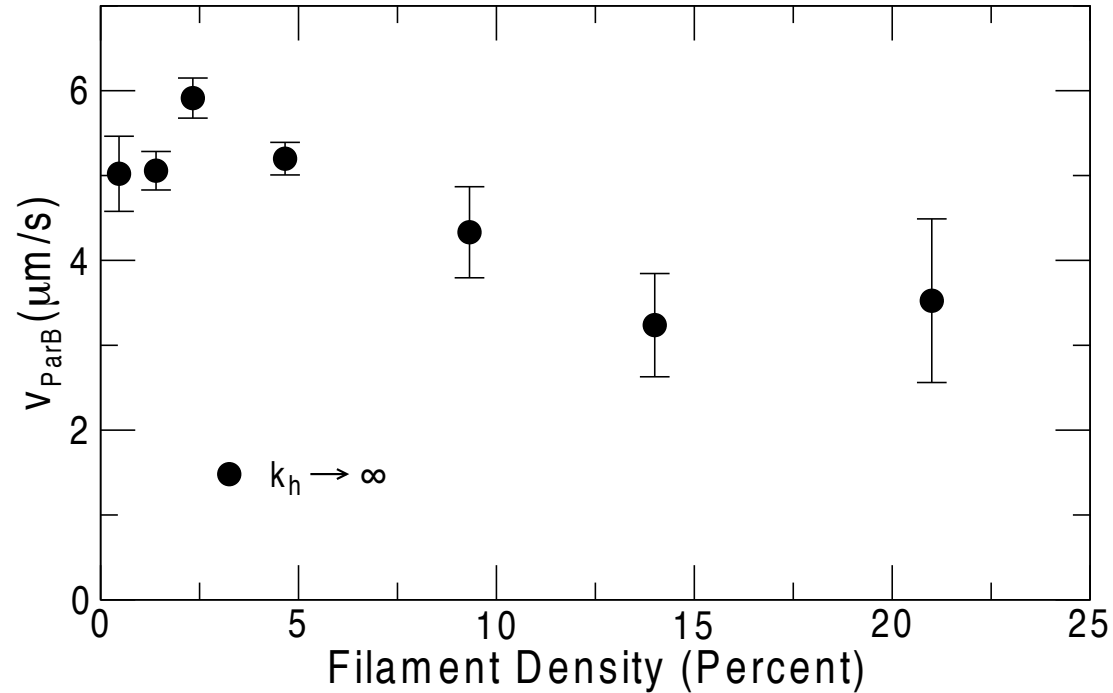

Supplement: Figure S2 — Dependence of translocation velocity, , on the density of ParA filaments within the ParA bundle. For ParA bundles of equal diameter, , but different numbers of ParA filaments, the translocation velocities are approximately equal. Thus, is insensitive to the density of filaments in the ParA bundle. (PDF) [file pcbi.1002145.s002.pdf]

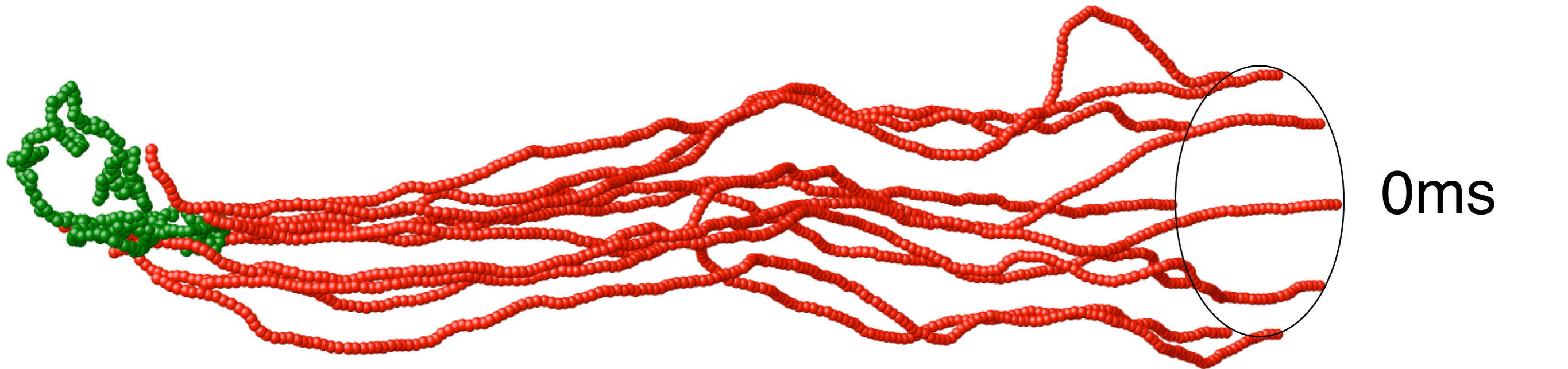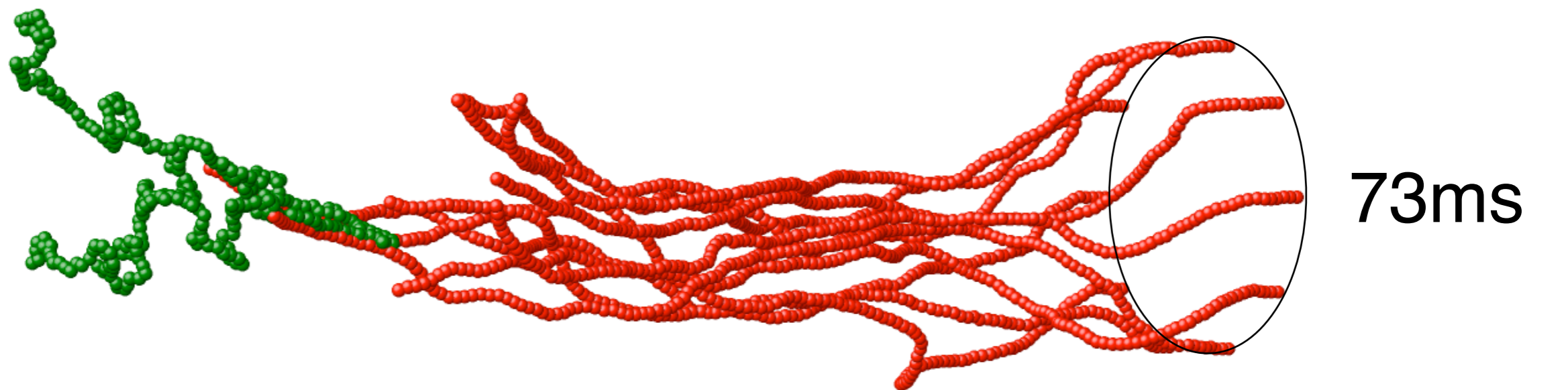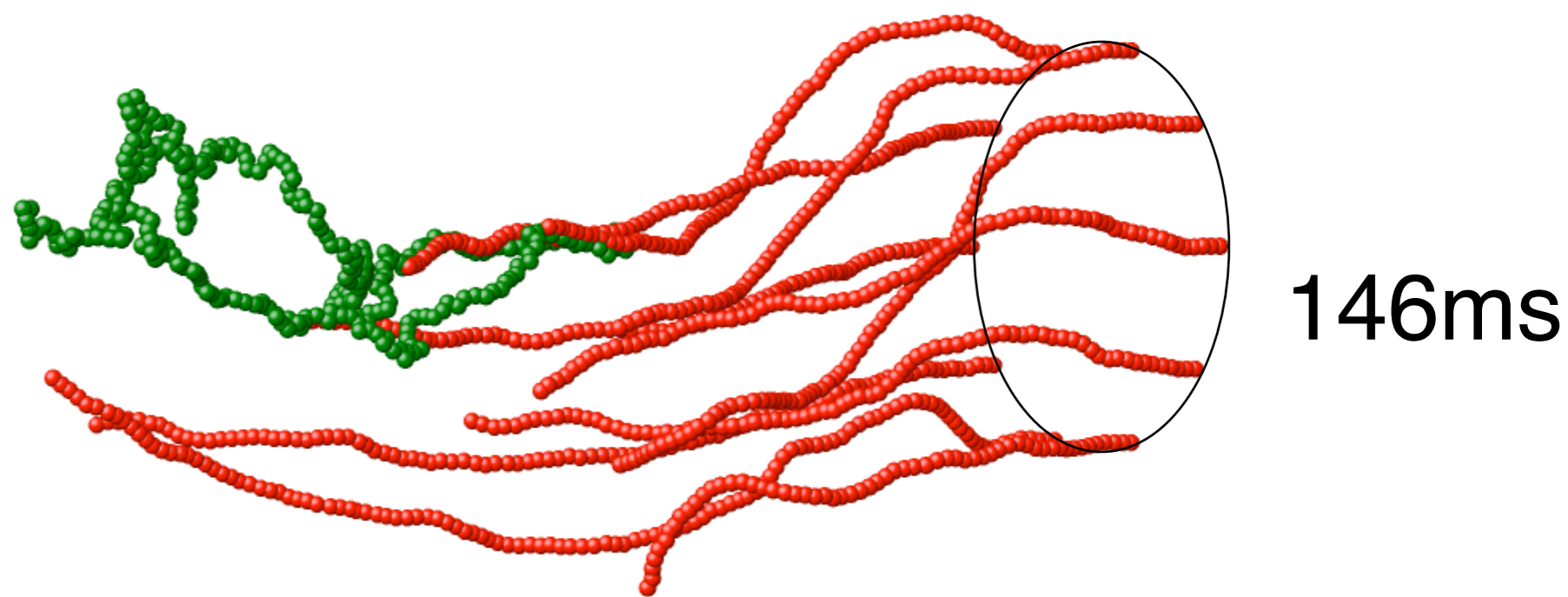

Supplement: Figure S3 — Snapshots of a simulation with a “ParA tube”. The ParA filaments in the ParA bundle are arranged cylindrically. The snapshots are slightly rotated into the page and the thin black circle indicates the base of the cylinder. Translocation of the ParB polymer is insensitive to whether the ParA filaments are arranged as a tube or as a bundle. Depolymerized ParA monomers are not shown. (PDF) [file pcbi.1002145.s003.pdf]

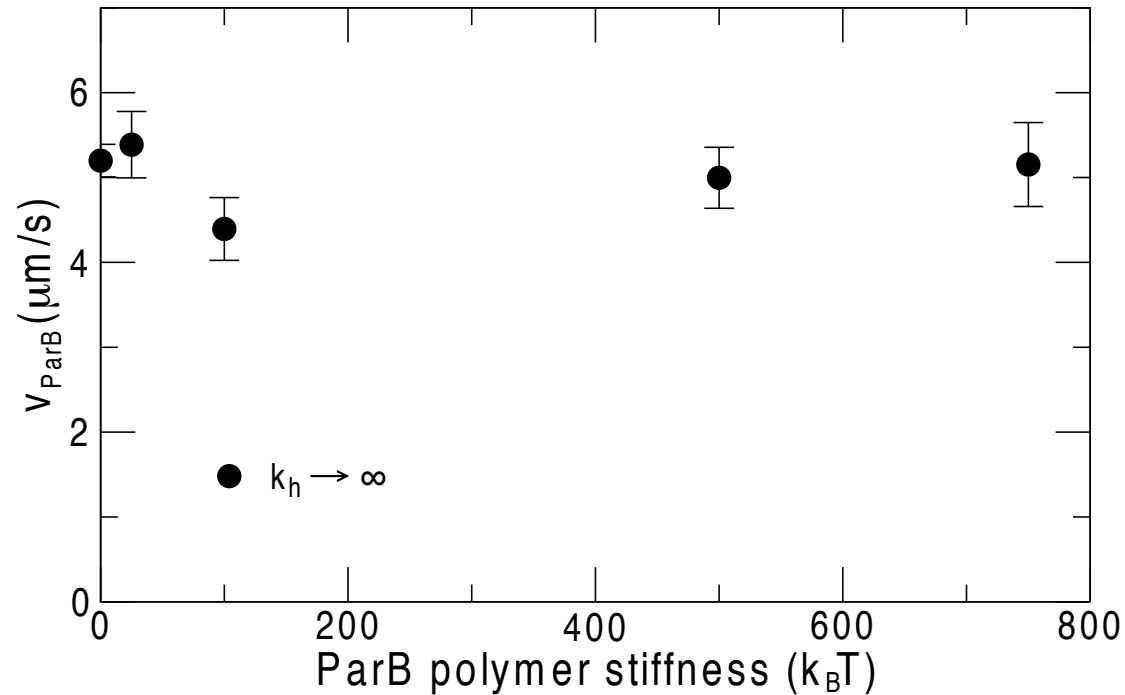

Supplement: Figure S4 — Dependence of translocation velocity, , on the stiffness of the ParB polymer. In our standard model, the ParB polymer is flexible, and the bending stiffness is . In order to simulate a stiff ParB polymer, we apply the bending potential in Eq. 11 to the ParB polymer. is insensitive to the bending stiffness over the observed range of . (PDF) [file pcbi.1002145.s004.pdf]

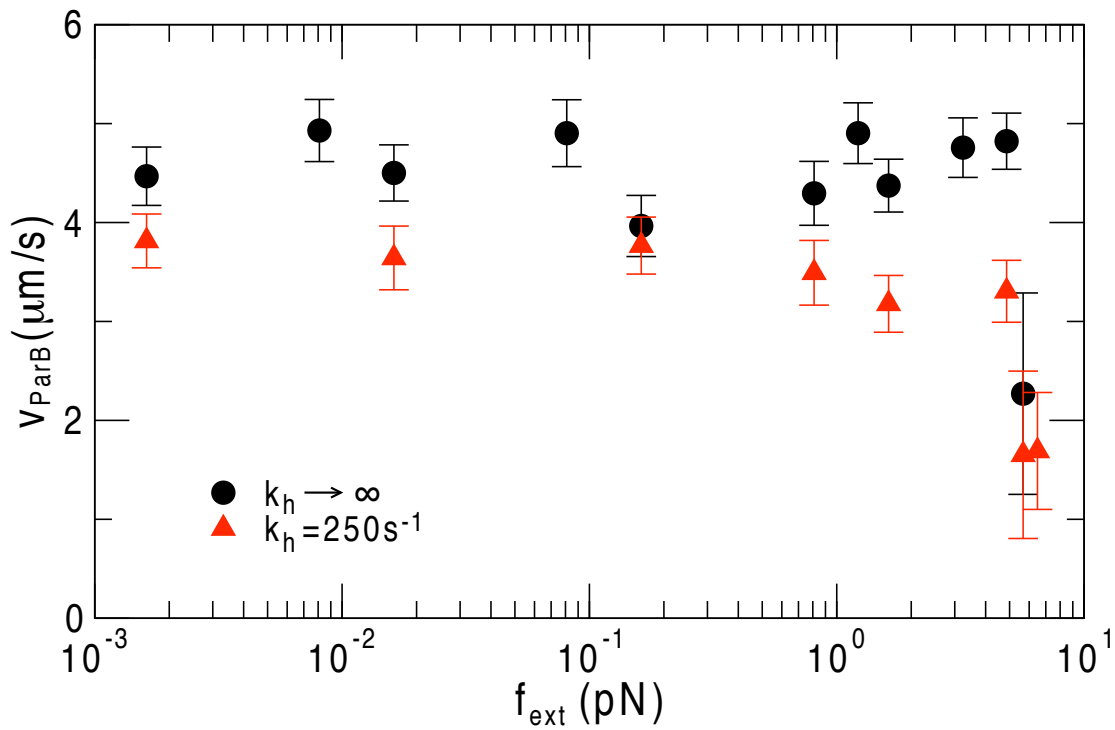

Supplement: Figure S5 — Force-velocity relation for ParB polymer translocation in our simulations. In these simulations, an external force, , pulls on each of the two ends of the ParB polymer, thus opposing depolymerization-driven translocation. Translocation of the ParB polymer is unperturbed when subjected to external pulling forces up to . (PDF) [file pcbi.1002145.s005.pdf]

ParA concentration

1  
0.5  
0

-50

0

50

100

150

Position (nm)

-- Tip-only-binding model  
-- Side-binding model

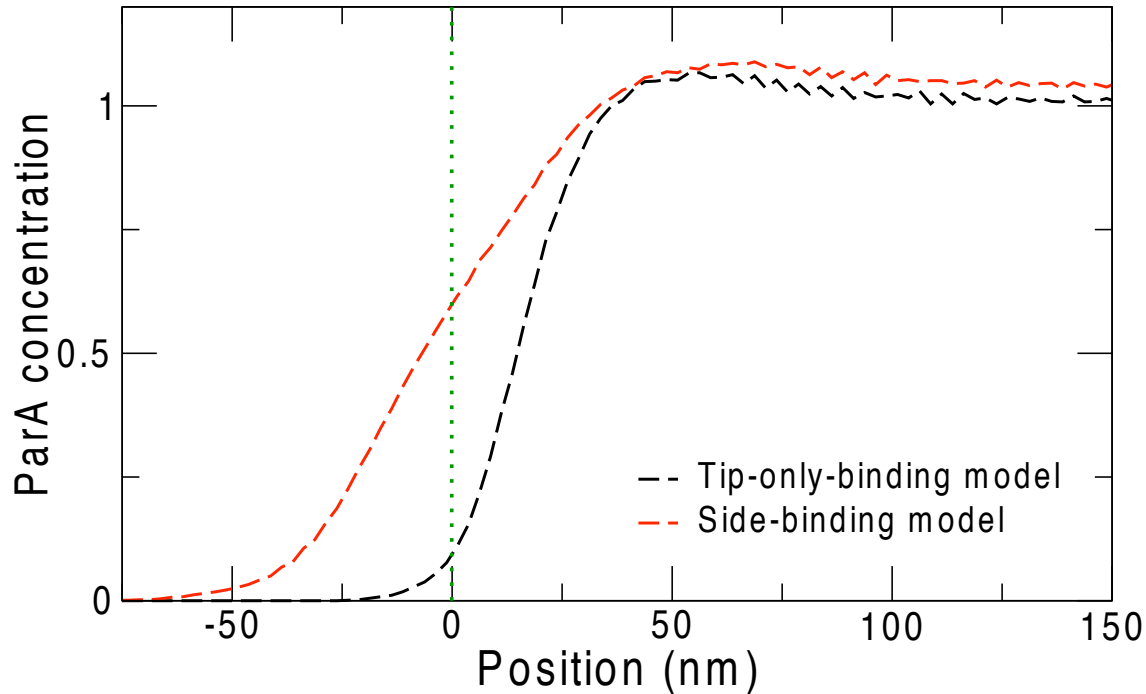

Supplement: Figure S6 — Steady-state ParA concentration profiles for tip-binding-only and side-binding models. Steady-state ParA concentration is plotted versus position relative to the center of mass of the ParB polymer, which is located at and indicated by the dotted green line. When ParB binds only to the tips of ParA filaments, the center of mass of the ParB polymer (dotted green line) localizes near the edge of the ParA filament concentration gradient (dashed black curve). This enables the ParB polymer to easily escape the ParA concentration gradient and detach from the ParA bundle due to thermal noise. However, when ParB can bind to the sides of ParA filaments, the ParB polymer penetrates further into the ParA bundle, and thus the center of mass (green) of the ParB polymer is localizes near the center of the ParA concentration gradient (dashed red curve). Thus, the ParB polymer is not susceptible to falling out of the ParA gradient and detaching from the ParA bundle due to thermal noise. (PDF) [file pcbi.1002145.s006.pdf]

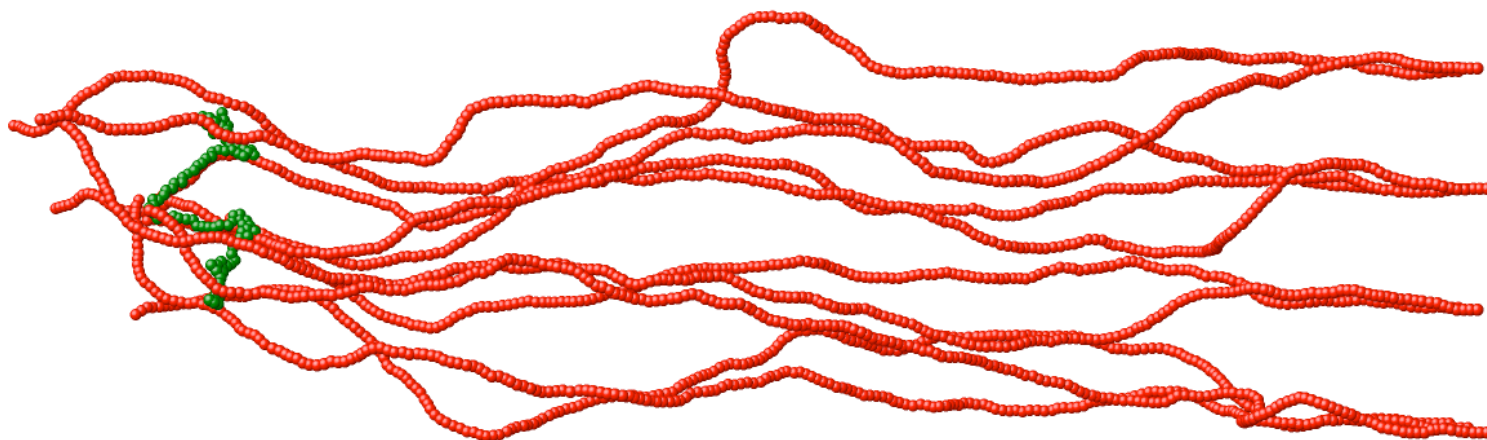

0ms

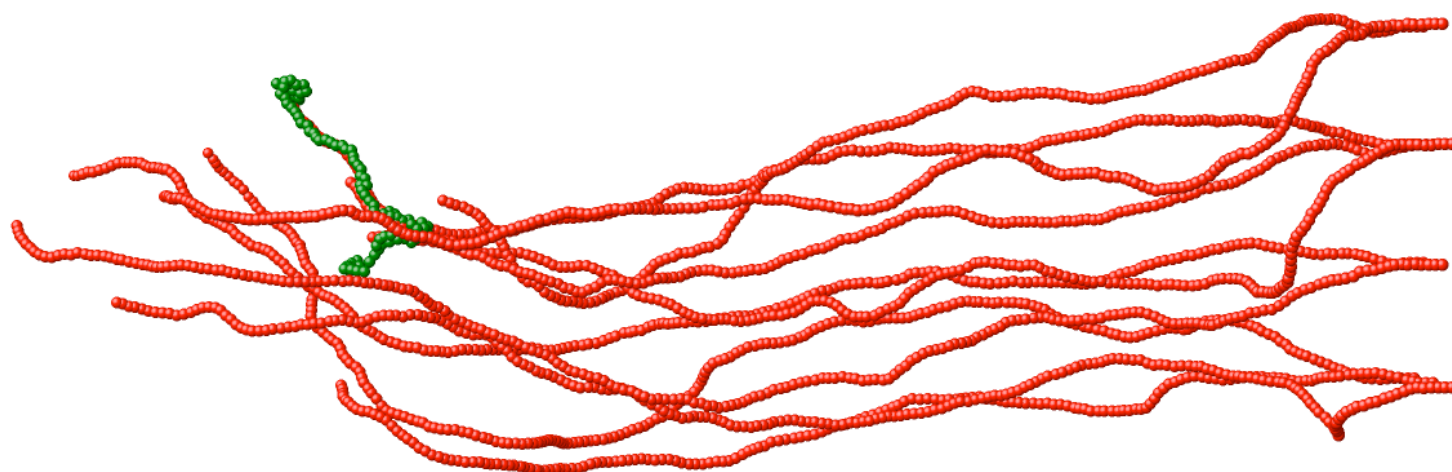

36ms

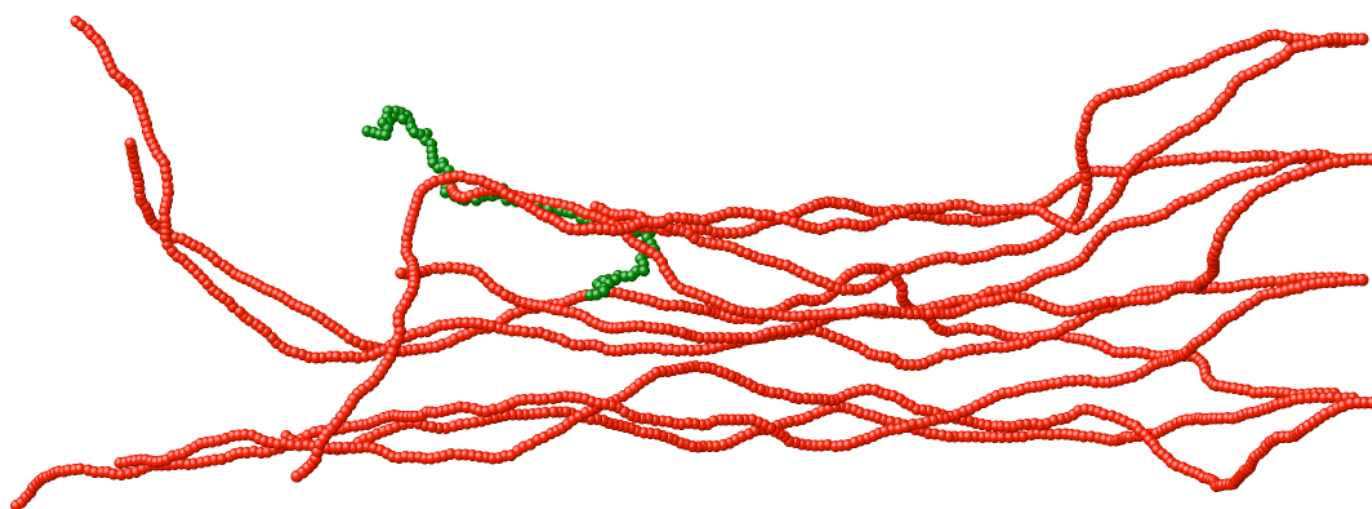

72ms

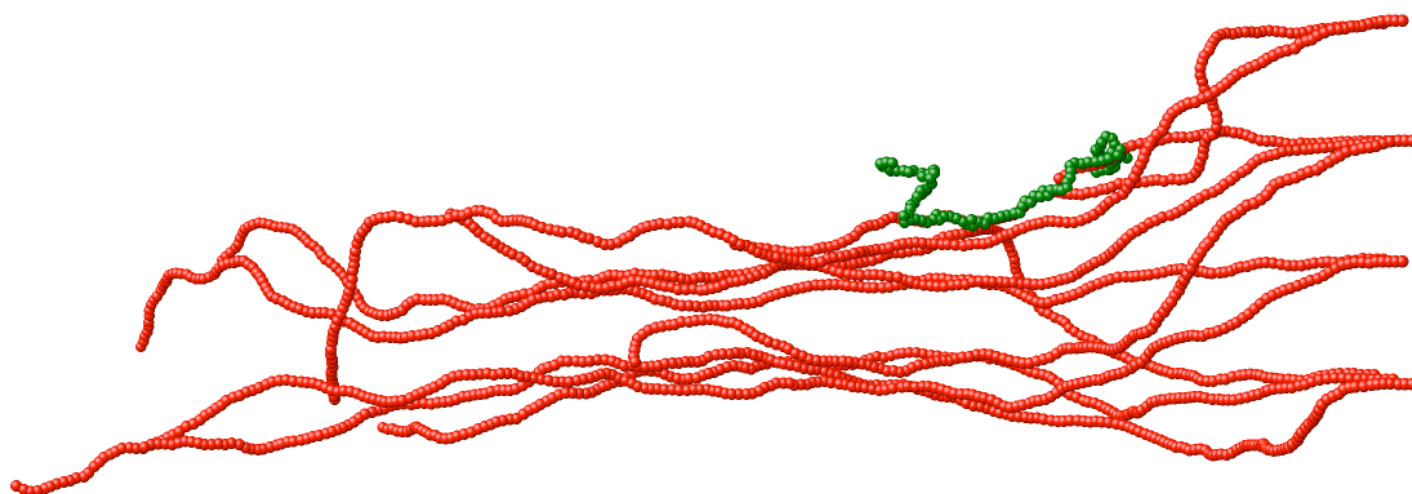

108ms

Supplement: Figure S7 — Snapshots of a simulation in which several ParA filaments remain after the ParB polymer has translocated. If the initial spacing, , of the ParA filaments in the bundle is large, the ParB polymer may translocate by disassembling some, but not all, of the ParA filaments. In the snapshots shown, the initial ParA filament spacing is , four times greater than the initial spacing, used in our standard simulations. This simulation demonstrates the versatility of our model by replicating one of the observations of Ptacin et al. (2010) [10]. This result can also be obtained with closely packed (e.g., ) ParA filaments if the filament bundle contains a large number of filaments. (PDF) [file pcbi.1002145.s007.pdf]
