# Supplementary material for: Filament Depolymerization Can Explain Chromosome Pulling during Bacterial Mitosis
Source: PLoS Comput Biol. 2011 Sep 22;7(9):e1002145. doi: 10.1371/journal.pcbi.1002145 (PMC3178632; doi:10.1371/journal.pcbi.1002145)
Supplement: Text S1 — Polymer relaxation time and estimated detachment force for the ParB polymer. This text explains how to calculate the characteristic polymer relaxation time, , and the peripheral segment diffusion coefficient, . In addition, we provide details for the estimation of the detachment force, . (PDF) [file pcbi.1002145.s008.pdf]

Filament depolymerization can explain chromosome pulling during bacterial mitosis: Text S1

Edward J. Banigan[1], Michael A. Gelbart[2,3], Zemer Gitai[4], Ned S. Wingreen[4,*], Andrea J. Liu[1,*]
1 Department of Physics and Astronomy, University of Pennsylvania, Philadelphia, PA, USA
2 Graduate Program in Biophysics, Harvard University, Boston, MA, USA
3 Department of Physics, Princeton University, Princeton, NJ, USA
4 Department of Molecular Biology, Princeton University, Princeton, NJ, USA
$\ast$ E-mail: ajliu@physics.upenn.edu (AJL); wingreen@princeton.edu (NSW)

Polymer Relaxation Time

The characteristic relaxation time of a polymer is typically given by the square of the characteristic length scale divided by the diffusion coefficient. Thus, the relaxation time of a peripheral segment, $\tau_r = (R_z^0)^2/D_s$, can be calculated by simulating a freely diffusing polymer segment and measuring $\langle (R_z^0)^2 \rangle$ and $\langle D_s \rangle$. $(R_z^0)^2$, the z-component of the equilibrium radius of gyration squared is found by calculating:

$$(R_z^0)^2 = \frac{1}{3}(R_g^0)^2 = \frac{1}{3N}\sum_{i=1}^{N}(\vec{r}_i - \vec{r}_{CM})^2 \tag{1}$$

where R_g^0 is the equilibrium radius of gyration, N is the number of subunits in the peripheral segment, $\vec{r}_i$ is the position of subunit i, and $\vec{r}_{CM}$ is the position of the center of mass of the peripheral segment. The diffusion coefficient of the peripheral segment, D_s, is calculated by measuring the mean squared displacement of the center of mass of the polymer over a given time interval:

$$D_s = \langle (\Delta r_{CM})^2 \rangle / 6\Delta t \tag{2}$$

where $(\Delta r_{CM})^2$ is the squared displacement of the center of mass after time interval Δt [1].

Estimated Detachment Force for the ParB Polymer

In the main text, we described the conditions under which intrinsic properties (i.e., k_0, τ_a, and ϵ) of the ParAB system do not support robust translocation (see Eq. 4 and Figs. 2-4 in the main text). Here we investigate the extent to which self-diffusiophoretic motility is robust to an external force on the chromosome that opposes translocation. In particular, we estimate the "detachment force," f^*, required to pull the ParB polymer off of the ParA bundle in a time, τ^*, that is approximately equal to the time required for the ParB polymer to translocate across the cell.

In our simulations, we model the ParB- *parS*- *ori* complex as a polymer chain comprised of N monomeric subunits. Each subunit in the central strip of the ParB polymer binds with a binding energy, ϵ, to a subunit in the ParA bundle. Thus, the total strength of the attraction between the ParB polymer and the ParA bundle is approximately proportional to $n\epsilon$, where n is the number of ParB subunits bound to ParA.

The number, n, of ParB subunits bound to ParA depends on the distribution, $p(z - z_{\mathrm{cm}})$, of ParB subunits about the center of mass of the polymer at z_{cm}. Here, z refers to the coordinate along the axis of the ParA bundle. We take the bundle to extend from $z = 0$ at the tip to $z = L$ at the swarmer pole (see Fig. 1c). For simplicity, we assume that the polymer is a Gaussian chain, so that the distribution along z is given by [1]:

$$p(z - z_{\mathrm{cm}}) = A \exp\left\{ -\frac{(z - z_{\mathrm{cm}})^2}{2R_z^2} \right\} \tag{3}$$

where R_z is the z-component radius of gyration of the polymer and $A = (2\pi R_z)^{-1/2}$ is the normalization constant.

We assume that every binding site of ParB that lies within the ParA bundle is bound to ParA. The number, $n = n(z_{\rm cm})$, of ParB subunits bound to the ParA bundle (which extends from $z = 0$ to $z = L$) is then given by the integral:

$$n(z_{\rm cm}) = N \int_0^L dz\ p(z - z_{\rm cm}) \approx N \int_0^\infty dz\ A \exp\left\{ -\frac{(z - z_{\rm cm})^2}{2R_z^2} \right\} \tag{4}$$

since $L \gg R_z$. Note that if only a fraction, ϕ, of ParB binding sites within the ParA bundle are bound, then n is simply reduced by the factor ϕ from the expression in Eq. S4.

Now consider the effect of a force $-f\hat{z}$ on the ParB polymer that opposes translocation in the $\hat{z}$ direction. At the simplest level, based on the above analysis, the ParB may be replaced by a point particle at the center of mass of the ParB polymer, $z_{\rm cm}$ in an effective potential given by

$$U(z_{\rm cm}) = -\epsilon n(z_{\rm cm}) + f z_{\rm cm}. \tag{5}$$

The first term is due to ParB binding to ParA and the second term is the work done by the external pulling force, f. As f increases, the minimum of U shifts to lower values of $z_{\rm cm}$ and the number of bound ParB sites decreases, eventually leading to unbinding of the ParB polymer from the ParA bundle.

The mean time for the particle to escape from the potential well (to detach from the ParA bundle) is well approximated by the Kramers escape time, $\tau_{\rm K}$ for this potential [2,3]:

$$\tau_{\rm K} = \frac{2\pi k_B T}{D} |U''(z_{\rm min}) U''(z_{\rm max})|^{-1/2} e^{(U(z_{\rm max}) - U(z_{\rm min}))/k_B T}. \tag{6}$$

The extrema of the potential are given by

$$0 = \frac{dU}{dz} = -\epsilon N A \exp\left\{ -\frac{z^2}{2R_z^2} \right\} + f \tag{7}$$

$$z_{\rm min/max} = \pm\sqrt{2R_z^2 \ln \frac{\epsilon N A}{f}}. \tag{8}$$

The second derivative is

$$\frac{d^2U}{dz^2} = \frac{\epsilon N A z}{R_z^2} \exp\left\{ -\frac{z^2}{2R_z^2} \right\}. \tag{9}$$

Given these expressions, we calculate the detachment force f^* to be the force f for which the escape time, τ_K, is equal to τ^*, the time required for the ParB polymer to translocate across the cell.

References

1. Doi M, Edwards SF (1986) The theory of polymer dynamics. Oxford: Clarendon Press, 14-16, 22-23, 95 pp.

2. Kramers HA (1940) Brownian motion in a field of force and the diffusion model of chemical reactions. Physica (Utrecht) 7: 284-304.

3. Risken H (1996) The Fokker-Planck quation. Berlin: Springer-Verlag, 96-99, 122-125 pp.

4. Ptacin JL, Lee SF, Garner EC, Toro E, Eckart M, et al. (2010) A spindle-like apparatus guides bacterial chromosome segregation. Nat Cell Biol 12: 791-798.

Supporting Figures

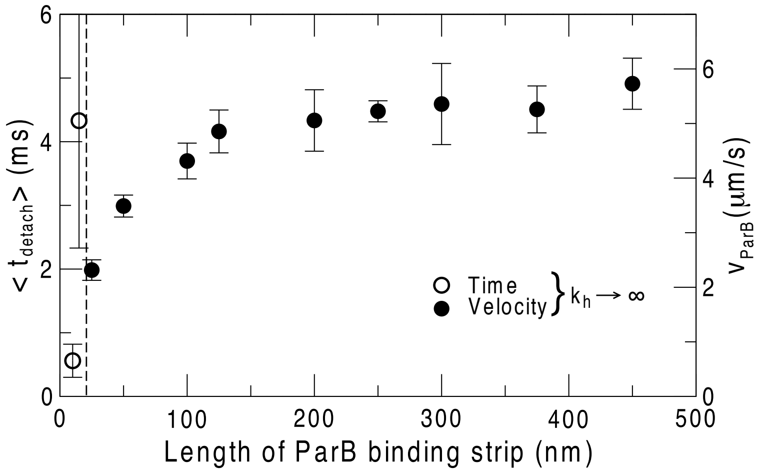

Figure S1. Behavior of the ParB polymer as a function of the length of the central ParB strip that binds to ParA. If too few of the ParB can bind to ParA, the ParB polymer detaches in an observably finite average time, $\langle t_{\mathrm{detach}} \rangle$ (open symbols). When the percentage of binding sites is above threshold, the translocation velocity, v_{ParB}, is non-zero. If there are enough binding sites to cause disassembly at all of the ParA filament tips simultaneously, v_{ParB} is insensitive to the number of ParB that can bind ParA. The dashed line separates the regimes of detachment and translocation.

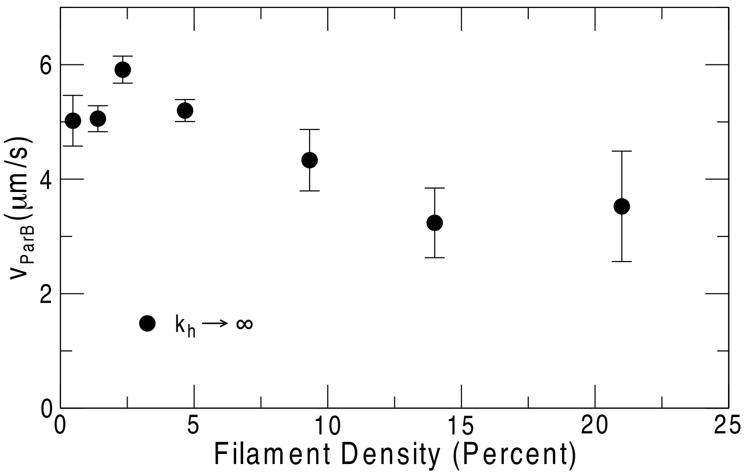

Figure S2. Dependence of translocation velocity, v_{ParB}, on the density of ParA filaments within the ParA bundle. For ParA bundles of equal diameter, $d \approx 6a$, but different numbers of ParA filaments, the translocation velocities are approximately equal. Thus, v_{ParB} is insensitive to the density of filaments in the ParA bundle.

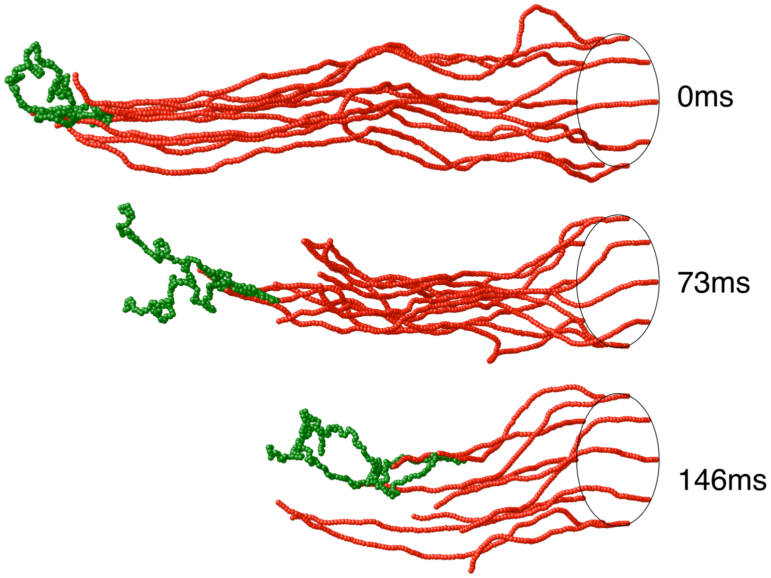

Figure S3. Snapshots of a simulation with a "ParA tube". The ParA filaments in the ParA bundle are arranged cylindrically. The snapshots are slightly rotated into the page and the thin black circle indicates the base of the cylinder. Translocation of the ParB polymer is insensitive to whether the ParA filaments are arranged as a tube or as a bundle. Depolymerized ParA monomers are not shown.

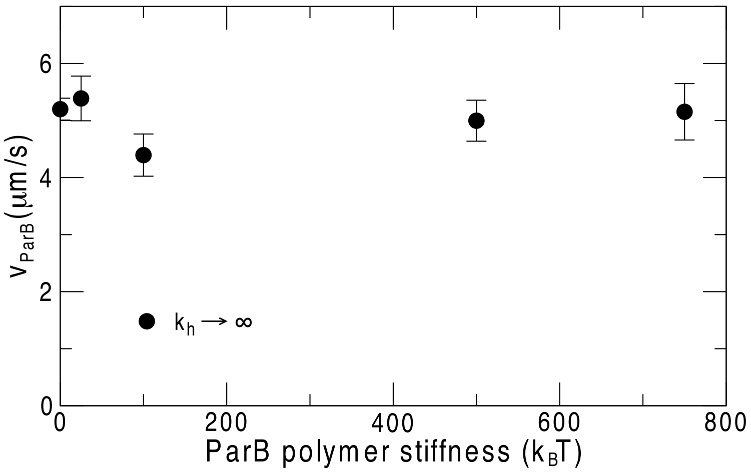

Figure S4. Dependence of translocation velocity, v_{ParB}, on the stiffness of the ParB polymer. In our standard model, the ParB polymer is flexible, and the bending stiffness is $K_S = 0k_BT$. In order to simulate a stiff ParB polymer, we apply the bending potential in Eq. 11 of the main text to the ParB polymer. v_{ParB} is insensitive to the bending stiffness over the observed range of K_S.

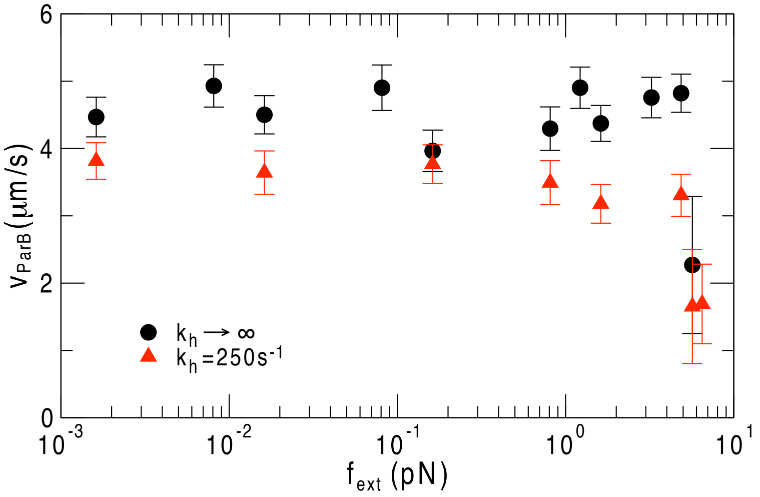

Figure 1. Force-velocity relation for ParB polymer translocation in our simulations. In these simulations, an external force, $f_{\mathrm{ext}}/2$, pulls on each of the two ends of the ParB polymer, thus opposing depolymerization-driven translocation. Translocation of the ParB polymer is unperturbed when subjected to external pulling forces up to $f_{\mathrm{ext}} \approx 7$pN.

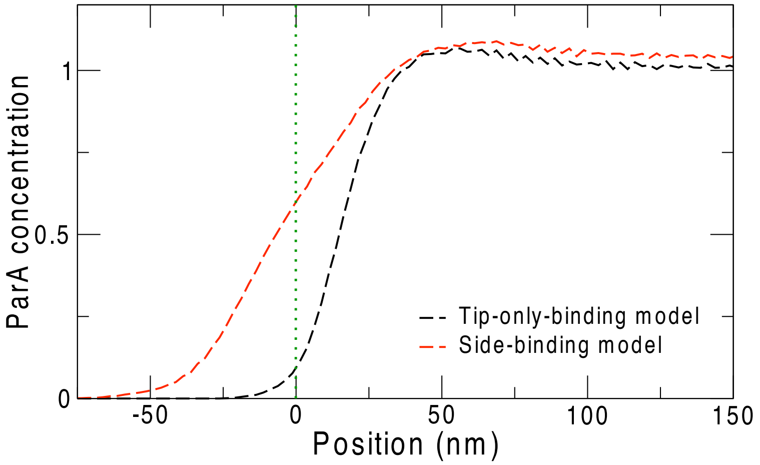

Figure S6. Steady-state ParA concentration profiles for tip-binding-only and side-binding models. Steady-state ParA concentration is plotted versus position relative to the center of mass of the ParB polymer, which is located at $z = 0$nm and indicated by the dotted green line. When ParB binds only to the tips of ParA filaments, the center of mass of the ParB polymer (dotted green line) localizes near the edge of the ParA filament concentration gradient (dashed black curve). This enables the ParB polymer to easily escape the ParA concentration gradient and detach from the ParA bundle due to thermal noise. However, when ParB can bind to the sides of ParA filaments, the ParB polymer penetrates further into the ParA bundle, and thus the center of mass (green) of the ParB polymer is localizes near the center of the ParA concentration gradient (dashed red curve). Thus, the ParB polymer is not susceptible to falling out of the ParA gradient and detaching from the ParA bundle due to thermal noise.

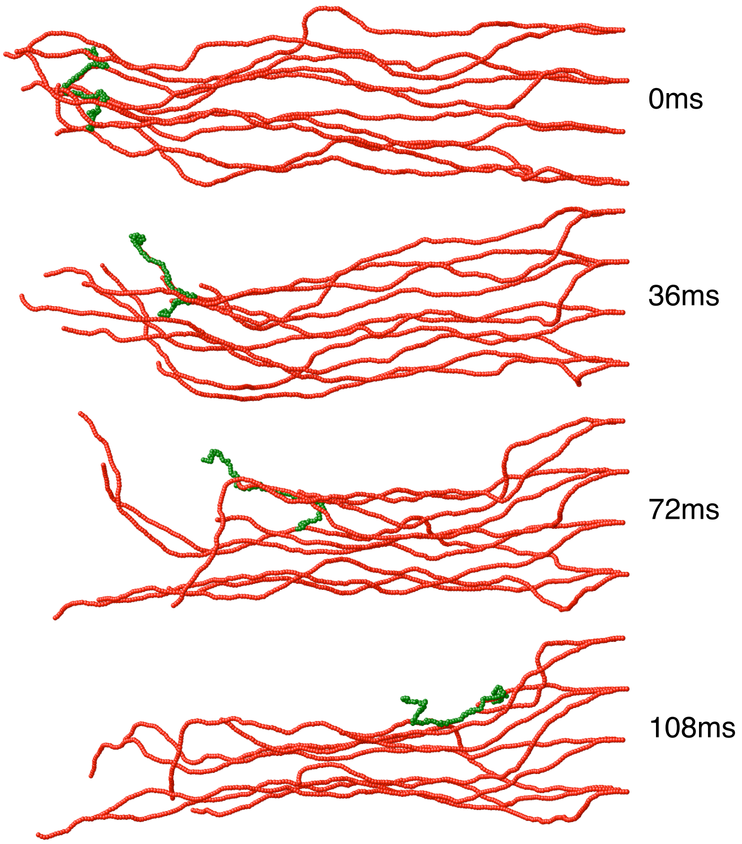

Figure S7. Snapshots of a simulation in which several ParA filaments remain after the ParB polymer has translocated. If the initial spacing, b, of the ParA filaments in the bundle is large, the ParB polymer may translocate by disassembling some, but not all, of the ParA filaments. In the snapshots shown, the initial ParA filament spacing is $b = 20a$, four times greater than the initial spacing, used in our standard simulations. This simulation demonstrates the versatility of our model by replicating one of the observations of Ptacin *et al.* (2010) [4]. This result can also be obtained with closely packed (*e.g.*, $b = 5a$) ParA filaments if the filament bundle contains a large number of filaments.

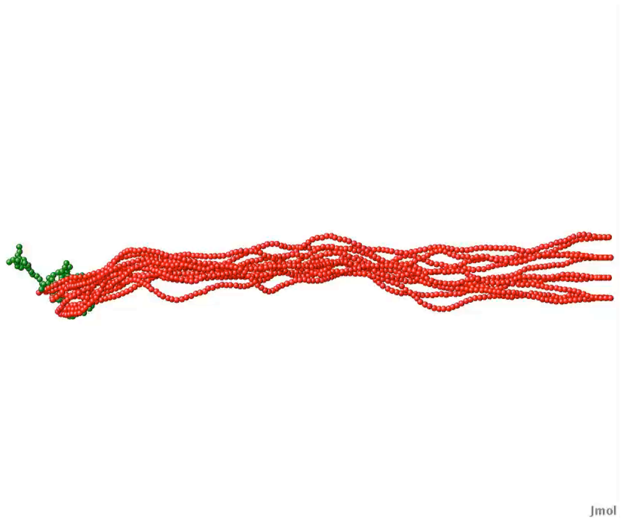

Video S1. A movie of translocation of the ParB polymer in our standard simulation conditions. The ParB polymer remains localized near the tip of the ParA bundle and translocates as the ParA bundle disassembles. Depolymerized ParA monomers are not shown.

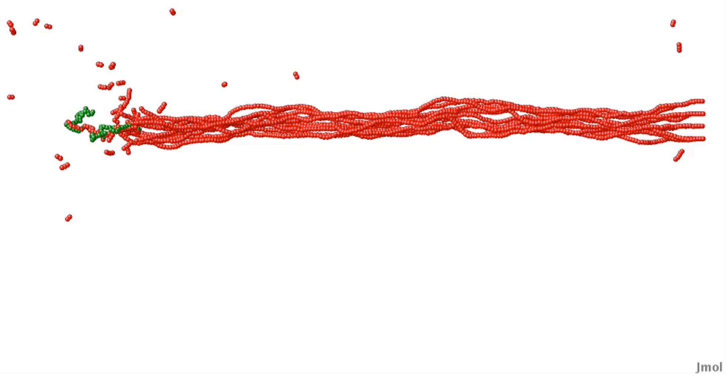

Video S2. A movie of a simulation run for the model in which ParB binds to the sides of ParA filaments and severs them. The ParB polymer translocates briefly until severed ParA protofilaments bind to the ParB polymer and disrupt its binding to the main ParA filament bundle.

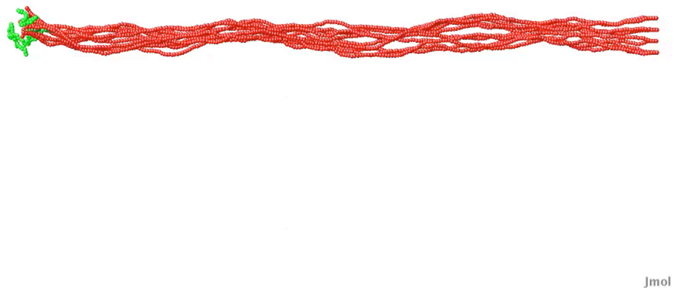

Video S3. Stretching of the ParB polymer. When the maximum ParA disassembly rate, k_0, is sufficiently large, the ParB polymer does not have time to relax to its equilibrium shape as it is pulled, and therefore stretches out. The ParB polymer consists of three segments; the two peripheral segments (light green), which cannot bind to ParA, and the central segment (dark green), which can bind to ParA. Note that the peripheral segments of the ParB polymer stretch, while the central segment of the ParB polymer is initially bound to the ParA filament bundle. When the peripheral segments stretch too far, they start to stretch the central segment, thus decreasing the length, ℓ, that the ParB polymer penetrates into the ParA bundle. This can lead to detachment for sufficiently high k_0. In this movie, $k_0 = k_d = 6170\text{s}^{-1}$, five times greater than the ParA disassembly rate, k_0, in our standard simulations (see Fig. 3 caption).
